# An Allelic Series of Mice Reveals a Role for RERE in the Development of Multiple Organs Affected in Chromosome 1p36 Deletions

**DOI:** 10.1371/journal.pone.0057460

**Published:** 2013-02-25

**Authors:** Bum Jun Kim, Hitisha P. Zaveri, Oleg A. Shchelochkov, Zhiyin Yu, Andrés Hernández-García, Michelle L. Seymour, John S. Oghalai, Fred A. Pereira, David W. Stockton, Monica J. Justice, Brendan Lee, Daryl A. Scott

**Affiliations:** 1 Department of Molecular and Human Genetics, Baylor College of Medicine, Houston, Texas, United States of America; 2 Department of Pediatrics, The University of Iowa, Iowa City, Iowa, United States of America; 3 Huffington Center on Aging and Department of Molecular and Cellular Biology, Baylor College of Medicine, Houston, Texas, United States of America; 4 Department of Otolaryngology-Head and Neck Surgery, Stanford School of Medicine, Stanford, California, United State of America; 5 Department of Otolaryngology–Head and Neck Surgery, Baylor College of Medicine, Houston, Texas, United States of America; 6 Departments of Pediatrics and Internal Medicine, Wayne State University School of Medicine, Detroit, Michigan, United States of America; 7 Howard Hughes Medical Institute, Baylor College of Medicine, Houston, Texas, United States of America; 8 Department of Molecular Physiology and Biophysics, Baylor College of Medicine, Houston, Texas, United States of America; Leibniz Institute for Age Research - Fritz Lipmann Institute (FLI), Germany

## Abstract

Individuals with terminal and interstitial deletions of chromosome 1p36 have a spectrum of defects that includes eye anomalies, postnatal growth deficiency, structural brain anomalies, seizures, cognitive impairment, delayed motor development, behavior problems, hearing loss, cardiovascular malformations, cardiomyopathy, and renal anomalies. The proximal 1p36 genes that contribute to these defects have not been clearly delineated. The arginine-glutamic acid dipeptide (RE) repeats gene (*RERE*) is located in this region and encodes a nuclear receptor coregulator that plays a critical role in embryonic development as a positive regulator of retinoic acid signaling. *Rere*-null mice die of cardiac failure between E9.5 and E11.5. This limits their usefulness in studying the role of RERE in the latter stages of development and into adulthood. To overcome this limitation, we created an allelic series of RERE-deficient mice using an *Rere*-null allele, *om*, and a novel hypomorphic *Rere* allele, *eyes3* (c.578T>C, p.Val193Ala), which we identified in an N-ethyl-N-nitrosourea (ENU)-based screen for autosomal recessive phenotypes. Analyses of these mice revealed microphthalmia, postnatal growth deficiency, brain hypoplasia, decreased numbers of neuronal nuclear antigen (NeuN)-positive hippocampal neurons, hearing loss, cardiovascular malformations–aortic arch anomalies, double outlet right ventricle, and transposition of the great arteries, and perimembranous ventricular septal defects–spontaneous development of cardiac fibrosis and renal agenesis. These findings suggest that RERE plays a critical role in the development and function of multiple organs including the eye, brain, inner ear, heart and kidney. It follows that haploinsufficiency of *RERE* may contribute–alone or in conjunction with other genetic, environmental, or stochastic factors–to the development of many of the phenotypes seen in individuals with terminal and interstitial deletions that include the proximal region of chromosome 1p36.

## Introduction

Telomeric deletions of the short arm of chromosome 1 are the most common telomeric deletions in humans–with an incidence of approximately 1 in 5000–and may account for up to 1.2% of idiopathic cognitive impairment [1]–[3]. In addition to cognitive impairment, children with 1p36 deletions can have eye anomalies, postnatal growth deficiency, brain anomalies, seizures, delayed motor development, behavioral problems, hearing loss, cardiovascular malformations, cardiomyopathy and renal anomalies [1], [4]. Early cytogenetic studies suggested that the genes responsible for most of these individual phenotypes were located in the distal region of chromosome 1p36 [5]. However, similar phenotypes–including cognitive impairment, postnatal growth deficiency, brain anomalies, seizures, delayed motor development, hearing loss, cardiovascular defects, and cardiomyopathy–have been seen in individuals with isolated interstitial deletions involving a non-overlapping section of the proximal region of chromosome 1p36 [6], [7]. The smallest reported deletion of this region spans ∼2.9 Mb [6]. This minimal deleted region could be considered a second contiguous gene deletion segment for the 1p36 deletion syndrome. Alternatively, it has been suggested that deletions of this region may be responsible for a clinically distinct genetic syndrome–proximal 1p36 deletion syndrome [6]. Since there is marked variability in the size of terminal 1p36 deletions, large deletions may involve both the distal and proximal regions of 1p36 [2], [8]. In such cases, it is likely that genes from both regions contribute to the associated phenotypes.

Several genes located in the distal region of 1p36 are thought to contribute to specific phenotypes seen in individuals with 1p36 deletions. These include *GABRD*, *PRKCZ*, *SKI*, and *KCNAB2* for neurodevelopmental anomalies and/or seizures, *SKI* for dysmorphic features and facial clefting, *GNB1* for behavioral abnormalities, and *MMP23A* and *MMP23B* for late closure of the anterior fontanel [9]–[13]. In some cases, mouse models provided evidence for the roles of these genes in specific 1p36 phenotypes. For example, *Gabrd* ^−/−^ and *Kcnab2* ^−/−^ mice have seizures and abnormal neurobehavioral phenotypes, *Gnb1* ^−/−^ mice have neural tube defects and impaired neural progenitor cell proliferation, and *Ski* ^+/−^ mice have midline facial clefts, neural tube defects and depressed nasal bridges [10], [14]–[16]. In contrast, the proximal 1p36 genes that contribute to specific phenotypes have yet to be identified.

One of the genes located in the proximal region of 1p36 is the arginine-glutamic acid dipeptide (RE) repeats gene (*RERE*) which is named after the dipeptide repeats contained within its carboxyl terminal. *RERE* was previously referred to as *ATN2* (Atrophin-2) due to its homology with *ATN1* (Atrophin-1). Although RERE is highly homologous to the C-terminal region of ATN1, with 67% amino acid sequence homology, RERE has distinctive BAH, EML2, SANT, and GATA domains in its N-terminal region [17].

Several lines of evidence suggest that RERE acts as a nuclear receptor coregulator. RERE has been shown to interact with histone deacetylase 1 (HDAC1*) in vitro* and in mouse embryos through its N-terminal region [18]. In glutathione S-transferase (GST) pull-down assays, Wang and colleagues demonstrated interactions between RERE and members of nuclear receptor subfamily 2 (NR2) such as NR2F (COUP-TF) and NR2E1 (TLX) [19].

Vilhais-Neto and colleagues went on to show that RERE forms a complex with NR2F2 (COUP-TFII), EP300 and a retinoic acid receptor [20]. This complex is recruited to the retinoic acid regulatory element of target genes after retinoic acid treatment [20]. Knockdown of either RERE or NR2F2 in NIH3T3 cells resulted in decreased activation of a retinoic acid response element (RARE)-luciferase reporter suggesting that this complex acts to promote the transcriptional activation of retinoic acid targets [20]. The role of RERE as a positive regulator of retinoic acid signaling was further demonstrated in F9 embryonal carcinoma cell lines treated with retinoic acid in which transfection with increasing amounts of an *Rere* expression vector resulted in increased retinoic acid signaling activity [20].

To date, mutations in *RERE* have not been implicated as the cause of a specific disease or syndrome in humans. However, the function of RERE in development has been explored using mouse models. In a recessive screen for mutations causing developmental defects in the forebrain, Zoltewicz and colleagues identified an N-ethyl-N-nitrosourea (ENU)-induced splice junction mutation in *Rere* (c.396+2T>A) that causes skipping of the second coding exon. This mutation was predicted to be a null allele based on virtually undetectable levels of mRNA by whole-mount *in situ* hybridization using a 5′ probe and failure to produce an in-frame transcript [18]. While mice that are heterozygous for this allele were viable and fertile, homozygous embryos died between E9.5 and E11.5. Early embryonic death was attributed to failure of cardiac looping and subsequent cardiac failure. These embryos also had defects in somitogenesis, fusion of the telencephalic vesicles, defects of the optic vesicles and failure of anterior neural tube closure for which they were given the name *openmind* (*om*) [18], [20]. In *Rere* ^om/om^ embryos, *Shh* expression failed to initiate along the anterior midline at E8.0, and *Fgf8* was delocalized from the anterior neural ridge at E8.5, revealing a crucial role for RERE in the formation and function of these two signaling centers [18].

Decreased retinoic acid signaling was subsequently seen in the trunk and forebrain regions of *Rere* ^om/om^ embryos bearing an RARE-lacZ reporter element–a sensitive reporter for the presence of endogenous retinoic acid [20]. This suggests that loss of RERE interferes with retinoic acid signaling in the mouse embryos and that abnormalities in retinoic acid signaling could play a role in the development of the phenotypes seen in *Rere* ^om/om^ embryos [20].

The early lethality seen in *Rere* ^om/om^ embryos limits their usefulness in studying the role of RERE in latter stages of development and into adulthood. To overcome this limitation, we created an allelic series of RERE-deficient mice using the *om* allele and a novel hypomorphic *Rere* allele, *eyes3* (c.578T>C, p.Val193Ala; MGI: 3577417), which we identified in an ENU-based screen for autosomal recessive phenotypes based on the presence of microphthalmia [21]. In addition to microphthalmia, *Rere* ^om/eyes3^ mice have a high level of perinatal mortality, postnatal growth deficiency, brain hypoplasia, decreased numbers of neuronal nuclear antigen (NeuN)-positive hippocampal neurons, hearing loss, cardiovascular malformations, spontaneous development of cardiac fibrosis in adulthood and renal agenesis. These findings suggest that RERE plays a critical role in the development and function of multiple organs including the eye, brain, inner ear, heart and kidney and that haploinsufficiency of *RERE* may contribute–alone or in conjunction with other factors–to the development of many of the phenotypes seen in individuals with deletions that include the proximal region of 1p36.

## Materials and Methods

### Ethics Statement

All experiments using mouse models were conducted in accordance with the recommendations in the Guide for the Care and Use of Laboratory Animals of the National Institutes of Health. The associated protocols were approved by the Institutional Animal Care and Use Committee of Baylor College of Medicine (Animal Welfare Assurance #A3832-01).

All efforts were made to minimize suffering. Experiments that were likely to induce discomfort, distress, pain, or injury, or required the use of restraining devices were performed on mice that had been properly anesthetized using an intraperitoneal injection of ketamine (100 mg/kg) and xylazine (5 mg/kg). Euthanasia was carried out using methods consistent with the recommendations of the Panel of Euthanasia of the American Veterinary Medical Association and included carbon dioxide inhalation or an overdose of an inhaled anesthetic, such as isoflurane, in an appropriate enclosure.

### N-ethyl-N-nitrosourea (ENU) Mutagenesis and Generation of *eyes3* Mice

ENU mutagenesis was carried out using 8 to 12 week male C57BL/6Brd^Tyr^ mice given 300 mg of N-ethyl-N-nitrosourea per kg of body weight administered in three 100 mg/kg intraperitoneal injections at 1-week intervals as previously described [22]. These mice were then bred and intercrossed to screen for viable recessive phenotypes. The *eyes3* strain (MGI: 3577417) was identified based on the presence of microphthalmia and decreased body size [21].

### Mapping and Cloning

Mice from the *eyes3* strain were backcrossed to 129S6/SvEvTac mice and progeny were intercrossed to identify mice carrying the homozygous *eyes3* allele based on the presence of microphthalmia. After several generations of backcrossing, *eyes3* mice were genotyped using single nucleotide polymorphism and microsatellite markers that discriminate between C57BL/6Brd^Tyr^ and 129S6/SvEvTac strains. Linkage analysis was performed as described previously [23]. The mutation responsible for the *eyes3* phenotype was localized to mouse chromosome 4 and haplotype analysis revealed that the mutation resided within an approximately 2.2 Mb interval between markers D4MIT362 (148716333–148716521, NCBIM37) and D4MIT42 (150944103–150944202, NCBIM37).

*Rere* was selected for further analysis based, in part, on the results of phylogenetic profiling as previously described [24]. Briefly, candidate genes from the interval were prioritized by comparative phylogenetic analysis using the method described by Chiang and colleagues [25]. We compiled two gene sets which were compared against 14 proteomes representing different branches of the phylogenetic tree. Gene set 1 included candidate genes from the linkage interval. Gene set 2 included mouse orthologues of genes previously shown to cause structural eye defects in *H. sapiens*. The proteome databases were obtained from NCBI (www.ncbi.nlm.nih.gov/; *S. cerevisiae, C. elegans, A. gambiae, D. melanogaster, C. intestinalis, X. tropicalis, D. rerio, M. musculus, H. sapiens*), EMBL-EBI (http://www.ebi.ac.uk/; *A. thaliana* strain *Columbia*, *D. discoideum,* strain *AX4*, and *E. coli* strain *K12*) and Ensembl (http://useast.ensembl.org/index.html; *G. gallus, O. anatinus).* The mouse protein sequences for gene set 2 were obtained from Ensembl build 36 (*Eya1, Eya2, Six3, Gli3, Pax6, Mks1, Tmem67 (Mks3), Pax2*, *Casp3, Sox2, Shh, Bcor, Chx10, Sox10*, *Otx2, Rax, Hesx1, Ikbkg, Ptch1, Pomt1,* RP23-464N23.1 *(Chd7*), *Gmnn, Mitf, Ndph, Cryba4, Crybb2).* Control and candidate gene sets were compared against proteomes using BLASTP 2.2.14 deployed on a PC platform. The returned e-values were analyzed with a custom written Perl script (ActivePerl 5.8.8 build 817). The enriched gene set was further prioritized based on the known or predicted role of each gene in organogenesis through comprehensive profiling.

The coding region and associated intron/exon junctions of *Rere* were amplified by PCR and the resulting amplification products were sequenced. Sequence traces were analyzed using Sequencher 4.7 software (Gene Codes Corporation, Ann Arbor, MI).

### Western Blot Analyses

Embryos for each genotype were homogenized with lysis buffer containing 20 mM Tris-HCl (pH7.5), 150 mM NaCl, 1 mM Na_2_EDTA, 1% Triton X-100, 2.5 mM sodium pyrophosphate, 1 mM Na_2_VO_4_, and Complete Protease Inhibitor Cocktail (Roche Applied Bioscience, Mannheim, Germany) per manufacturer’s instructions. Protein extracts prepared from homogenates were resolved by SDS-PAGE and transferred to nitro cellulose membranes. These membranes were probed with the following antibodies: anti-RERE (sc-98415, 1∶500; Santa Cruz Biotechnology, Santa Cruz, CA, USA) and anti-β-actin (#4970, 1∶1000; Cell Signaling, Danvers, MA, USA). Each blot was visualized using a SuperSignal West Pico Chemiluminescent detection kit (Thermo Scientific, Rockford, IL, USA) per manufacturer’s instructions and quantified using ImageJ software (http://rsbweb.nih.gov/ij/). The expression level of RERE was normalized by the intensity of the β-actin band in the blot. Data from three independent embryos of each genotype were used for quantification.

### Histological Analysis

#### Preparation of paraffin embedded tissue sections

Tissues were dissected out and fixed with Buffered Formalde-Fresh solution (Fisher Scientific, Pittsburgh, PA, USA) for 1 day at 4°C. After washing with phosphate buffered saline solution (PBS), tissues were dehydrated in ethanol and embedded in paraffin. Paraffin embedded tissue blocks were sectioned at 6 µm with an RM2155 microtome (Fisher Scientific).

#### Tissue staining

Nissl staining was used for histological analyses of the brain. Hematoxylin and eosin (H&E) staining was used for evaluation of cardiovascular malformations. For identification of fibrosis in the heart, paraffin embedded heart tissue sections were stained using a Masson’s Trichrome Stain Kit (Sigma-Aldrich, Saint Louis, MO, USA) after deparaffinization.

#### Immunohistochemical staining

After deparaffinizing, brain tissue sections were blocked with PBS containing 1% bovine serum albumin and 5% normal donkey serum and incubated with anti-RERE (sc-98415, 1∶100; Santa Cruz Biotechnology, Santa Cruz, CA, USA), anti-NeuN (MAB377, 1∶500; Millipore, Billerica, MA, USA), anti-Phospho-Histone H3 (pHH 3) (#9701, 1∶200; Cell Signaling, Danvers, MA), anti-Cleaved Caspase-3 (#9664, 1∶200; Cell Signaling, Danvers, MA, USA), anti-Nestin (MAB353, 1∶1000; Millipore, Billerica, MA, USA), anti-GFAP (AB5804, 1∶1000; Millipore, Billerica, MA, USA), or anti-Calretinin (ab702, 1∶200; Abcam, Cambridge, MA, USA) antibodies. This was followed by incubation with HRP conjugated anti-rabbit IgG or anti-mouse IgG (Jackson ImmunoResearch, West Grove, PA, USA). Immunoreactivity of each antibody was visualized using either a 3,3′-diaminobenzidine (DAB) substrate kit (Vector Laboratories, Burlingame, CA, USA) or a tyramide signal amplification (TSA) kit (Invitrogen, Grand Island, NY, USA).

### Neurobehavioral Tests

#### Open field activity tests

The locomotor activity of mice was measured by placing them in the center of a clear Plexiglas (40×40×30 cm) open field arena which they were allowed to explore for 30 min. Data were collected in 2 min intervals over the 30 min test time. Activity was recorded and quantitated using the Versamax animal activity monitoring system (Accusan Instruments, Columbus, OH, USA) which contains 16 photoreceptor beams on each side of the arena, effectively dividing the arena into 256 equally-sized squares. The testing room had overhead incandescent light bulbs which provided approximately 800 lux of light. White noise of approximately 55 dB was present throughout testing. Locomotor activity was measured by calculating the total distance the animal travelled during the 30 min test period. The level of anxiety-like behavior was determined using the center distance: total distance ratio as an index.

Open–field activity (locomotor activity) and the index indicating anxiety-like behavior were analyzed using one-way analysis of variance (ANOVA).

#### Rotarod tests

Motor coordination, balance and skill learning were tested using an accelerating Rotarod (UGO Basile, Comerio, VA, Italy). The Rotarod test assesses the ability of the animal to walk on the rotating rod which accelerates from 4 to 40 rpm over 5 min. Each animal had 8 trials–4 trials per day with an intertrial interval of at least 30 min. The learning index was calculated by subtracting the average time spent on the rod during the first two trials from the average time spent on the rod during the final two trials. Rotarod data were analyzed using a two-way (Training History × Trial) ANOVA with repeated measures.

#### Prepulse inhibition of acoustic startle

Prepulse inhibition and acoustic startle responses were measured using the SR-Lab System (San Diego Instruments, San Diego, CA, USA). Mice were acclimatized to the sound attenuating chamber by placing them in a cylindrical tube with a background noise of 70 dB for 5 min before the test session. The test consisted of 48 total trials with six blocks of eight trial types, each occurring in a random order. The eight trial types were (1) “no stimulus” to measure the baseline movement when no sound is present; (2) a “startle only” trial consisting of a 40 ms, 120 dB sound burst; (3) three “prepulse only” trials of a 20 ms, 74, 78 or 82 dB sound burst; and (4) three “prepulse inhibition” trials where one of the three prepulses (74, 78 or 82 dB) was presented 100 ms before the startle stimulus (40 ms, 120 dB). The inter-trial interval was 10–20 s. The startle response after each trail was measured as force changes in the holding tube and was recorded for 65 ms following the stimulus.

Percent prepulse inhibition (% PPI) was calculated using the following formula: 100– [(response to acoustic prepulse plus startle stimulus trials/startle response alone trials) ×100]. Therefore, a low % PPI would indicate a poor PPI or that the animal’s startle response is the same with or without the prepulse whereas a high % PPI would indicate a good PPI or that the animal’s response to the startle is subdued due to the prepulse stimulus compared to when the startle stimulus was present alone. Acoustic response amplitude and PPI were analyzed using one-way ANOVA.

#### Conditioned fear

Conditioned fear testing was performed as previously described [26]. The test chamber (Med Associates Inc., St. Albans, VT, USA) was surrounded by a photobeam detection system that recorded freezing responses in the animals and the bottom of the chamber contained a grid used to deliver a mild electric shock (0.75 mA). This two-day test was divided into two parts with the first day being the training day and the second day being the test day. On the training day, the animal was placed in the test chamber for 5 min and experienced 2 conditioned stimulus-unconditioned stimulus (CS-US) pairings in the following order: 2 min of no stimulus, 30 s of white noise at 80 dB (CS), 2 s mild foot shock (US), another 2 min of no stimulus followed by a CS-US pair. Freezing behavior was recorded every 10 s and responses to the foot shock (run, jump and vocalize) were noted.

Twenty-four hours later, the contextual test was done by placing the mouse in the chamber for 5 min without presenting them with the white noise or the foot shock and recording the freezing behavior every 10 s.

Another hour later, the animal was put back in the chamber and tested for cued fear (CS test) by presenting the white noise but changing the environmental context as previously described [26]. The 6 min CS test consisted of 3 min of no sound (Pre-CS phase) followed by a 3 min of white noise (CS phase). Freezing behavior was again recorded every 10 s. Freezing during the CS test was determined using the following formula: % freezing during CS phase - % freezing pre-CS phase. Percent freezing was analyzed using one-way ANOVA.

### Auditory Startle Response Testing

Freely moving mice of each genotype were subject to 10 random 108 dB sound bursts at 19.9 kHz emitted from a click box (MRC Institute of Hearing Research, Nottingham, UK). Freezing behavior was recorded after each sound burst. Data were analyzed using a one-way ANOVA.

### Auditory Brainstem Response Testing

Auditory brainstem responses (ABRs) were measured as previously described [27]. Briefly, 3-week-old mice (n = 4 per genotype) were anesthetized using an intraperitoneal injection of ketamine (100 mg/kg) and xylazine (5 mg/kg) and immobilized in a head holder. Normal body temperature was maintained throughout the procedure by placing the mice on a heating pad. An ear bar was inserted into the ear canal such that the tip of the microphone was within 3 mm of the tympanic membrane. Pure tone stimuli from 4 kHz to 48 kHz were generated using Tucker Davis Technologies System 3 digital signal processing hardware and software (Tucker Davis Technologies Alachua, FL, USA) and the intensity of the tone stimuli was calibrated using a type 4938 1/4″ pressure-field calibration microphone (Bruel and Kjar, Nærum, Denmark).

Response signals were recorded with subcutaneous needle electrodes inserted at the vertex of the scalp, the postauricular region (reference) and the back leg (ground). Auditory thresholds were determined by decreasing the sound intensity of each stimulus from 90 dB to 10 dB in 5 dB steps until the lowest sound intensity with reproducible and recognizable waves in the response was seen.

Mean hearing thresholds ± standard deviations (dB SPL) were plotted as a function of stimulus frequency (kHz). Student’s t-test was done at each frequency for statistical analyses. One-way ANOVA was done between genotype and ABR threshold for overall analyses as well.

## Results

### The *eyes3* Phenotype is Caused by a Single Amino Acid Change in the BAH Domain of RERE

**Figure 1:**
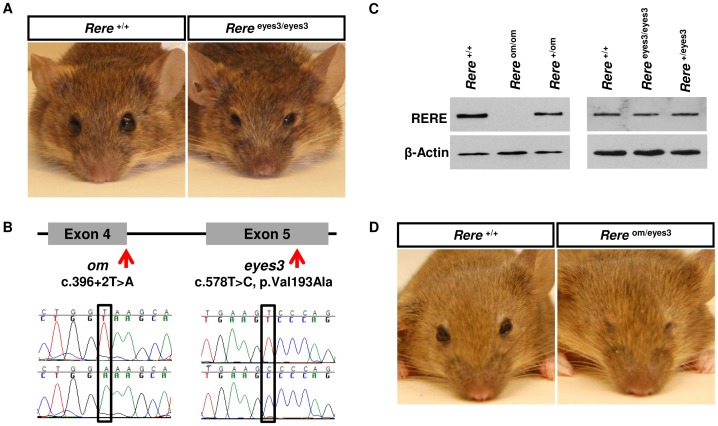
A hypomorphic mutation in *Rere* is responsible for microphthalmia in *eyes3* mice. A) *eyes3* mice were identified in an ENU screen for recessive mutations based on microphthalmia and were also found to have decreased body size and occasional unilateral renal agenesis. B) Sequencing of the *Rere* gene in *eyes3* mice revealed a homozygous missense mutation, c.578T>C, which codes for a single amino acid change in a highly conserved residue in RERE’s BAH domain (p.Val193Ala). The locations of the previously described *om* allele and the *eyes3* allele *Rere* are shown along with chromatograms from wild-type (top), *Rere* ^om/om^ (bottom left) and *Rere* ^eyes3/eyes3^ (bottom right) embryos. C) No discernible RERE protein is identified by western blot from *Rere* ^om/om^ embryos at E10.5 suggesting that the *om* allele is a null allele. In contrast, the expression of RERE protein is not affected in *Rere* ^eyes3/eyes3^ embryos at E10.5. This suggests that the deleterious effect of the p.Val193Ala change is caused primarily by reduced RERE function rather than decreased RERE protein expression. D) In contrast to *Rere* ^om/om^ embryos that die between E9.5 and E11.5, some *Rere* ^om/eyes3^ mice live into adulthood but have microphthalmia which is more severe than that seen in *Rere* ^eyes3/eyes3^ mice. These results suggest that the *eyes3* allele is a hypomorphic allele of *Rere*.

In an ENU-based screen for recessive mutations, we identified a line of mice, *eyes3*, with unilateral and bilateral microphthalmia (Fig. 1A), decreased body size (Fig. S1) and occasional unilateral renal agenesis [21]. The mutation responsible for the *eyes3* phenotype was mapped by linkage analysis to a region of mouse chromosome 4 that is syntenic to human chromosome 1p36.31–p36.22 and contained 25 validated or provisional RefSeq genes. Phylogenetic profiling of these genes and a review of the literature identified *Rere* as a potential candidate [18]. Sequencing of the coding region of *Rere* and associated intron/exon boundaries revealed a homozygous c.578T>C change which was predicted to cause a single amino acid change in a highly conserved residue in RERE’s BAH domain (p.Val193Ala) (Fig. 1B). This change was predicted to be “damaging” by SIFT (http://sift.jcvi.org/), a program designed to determine the probable consequences of single amino acid changes on protein function. No other sequence changes were identified.

The *om* allele of *Rere* (c.396+2T>A) is a splice junction mutation which causes skipping of the second coding exon of *Rere* and is predicted to be a null-allele–a prediction consistent with the lack of detectable RERE protein on western blot analyses from *Rere* ^om/om^ embryos at E10.5 (Fig. 1C) [18]. In contrast, western blot analyses detected comparable levels of RERE protein in *Rere* ^eyes3/eyes3^ embryos and wild-type controls at E10.5 (Fig. 1C, Fig. S2). This result suggests that the deleterious effect of the p.Val193Ala change is caused primarily by reduced RERE function rather than decreased RERE protein expression.

To confirm that the microphthalmia seen in the *eyes3* line was due to a defect in *Rere*, we conducted a complementation test by crossing heterozygous *om* mice (*Rere* ^+/om^) with heterozygous *eyes3* mice (*Rere* ^+/eyes3^). As expected, wild-type, *Rere* ^+/om^ and *Rere* ^+/eyes3^ progeny all had normal appearing eyes. In contrast, compound heterozygous *Rere* ^om/eyes3^ progeny had unilateral or bilateral microphthalmia (Fig. 1D). The presence of eye defects in *Rere* ^om/eyes3^ mice indicates failure of complementation between the *om* and *eyes3* alleles and confirms that the microphthalmia seen in *eyes3* mice is due to a defect in *Rere.*

### The *eyes3* Allele (c.578T>C, p.Val193Ala) is a Hypomorphic Allele of *Rere*

While both *Rere* ^eyes3/eyes3^ and *Rere* ^om/eyes3^ mice have unilateral or bilateral microphthalmia, these defects are generally more severe in *Rere* ^om/eyes3^ mice (Fig. 1A and 1D). This suggests that the *eyes3* allele is not as detrimental to RERE function as the *om* null-allele.

This pattern is also seen with regards to viability. In contrast to homozygous *om* embryos (*Rere* ^om/om^), which die between E9.5 and E11.5, *Rere* ^eyes3/eyes3^ mice are viable and fertile [18], [21]. As might be expected, *Rere* ^om/eyes3^ mice show an intermediate phenotype. In crosses between *Rere* ^+/om^ and *Rere* ^+/eyes3^ mice on a mixed B6Brd/129S6 background, *Rere* ^om/eyes3^ mice were recovered at Mendelian ratios at birth (P0, Table 1). However, the majority of the *Rere* ^om/eyes3^ pups died prior to weaning at P21.

**Table 1:** *Rere* ^om/eyes3^ mice have a high rate of perinatal mortality.

	*Rere* ^+/+^	*Rere* ^+/om^	*Rere* ^+/eyes3^	*Rere* ^om/eyes3^
**Adult (n = 268)** **	86 (32%)	88 (33%)	78 (29%)	16 (6%)
**P1 (n = 37)** *	11 (30%)	13 (35%)	10 (27%)	3 (8%)
**P0 (n = 64)**	17 (27%)	18 (28%)	13 (20%)	16 (25%)
**E17.5** **(n = 103)**	27 (26%)	26 (25%)	19 (18%)	31 (30%)

* *p* = 0.1,

** *p*<0.0001.

Taken together, these results suggest that the *eyes3* allele (c.578T>C, p.Val193Ala) is a hypomorphic allele of *Rere*.

### Postnatal Growth Deficiency in *Rere* ^om/eyes3^ Mice

**Figure 2:**
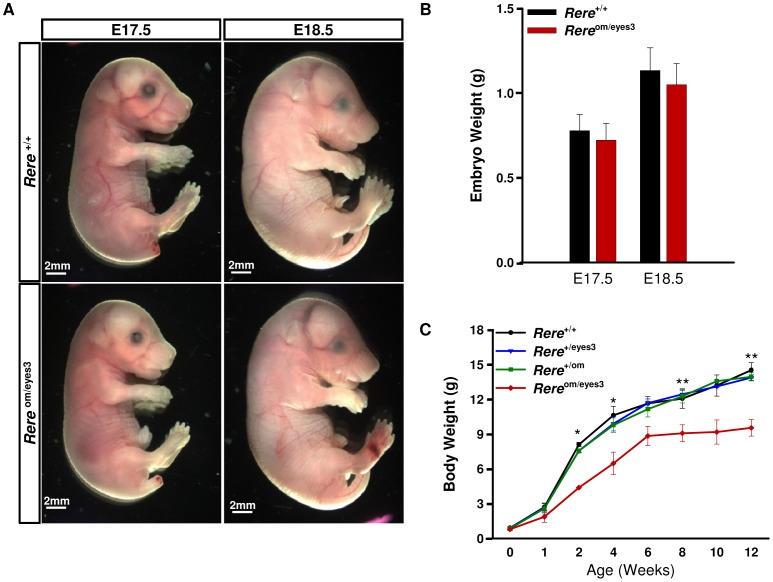
*Rere* ^om/eyes3^ embryos exhibit postnatal growth deficiency. A) The sizes of *Rere* ^om/eyes3^ embryos were comparable to wild-type embryos at E17.5 and E18.5. B) There was no significant difference in embryo weights between *Rere* ^om/eyes3^ embryos and their wild-type littermates at E17.5 and E18.5 (n≥4). C) Body weight was measured to evaluate the somatic growth of *Rere* ^om/eyes3^ mice between birth and 12 weeks of age. The body weights of *Rere* ^om/eyes3^ mice were significantly reduced after 1 week of age and plateaued at 6 weeks of age while wild-type, *Rere* ^+/eyes3^ and *Rere* ^+/om^ mice continued to gain weight (n = 5–10 for each genotype. * = p<0.01, ** = *p*<0.03).

Since children carrying terminal and proximal 1p36 deletions often have postnatal growth deficiency, and *Rere* ^eyes3/eyes3^ mice were noted to have decreased body size, we examined the somatic growth of *Rere* ^om/eyes3^ mice [1], [6], [7], [21]. At E17.5 and E18.5, the sizes and weights of *Rere* ^om/eyes3^ embryos were not statistically different from those of their wild-type littermates (Fig. 2A–B). At birth, the weights of *Rere* ^om/eyes3^ mice were not statistically different from those of their wild-type littermates but starting at one week of age (P7) they weighed significantly less than their wild-type littermates (Fig. 2C). At 6 weeks of age, the body weight of *Rere* ^om/eyes3^ mice reached a plateau while their control littermates continued to gain weight (Fig. 2C). These data suggest that RERE deficiency can cause postnatal growth deficiency in mice.

### Reduction of Brain Size and Weight in *Rere* ^om/eyes3^ Mice

**Figure 3:**
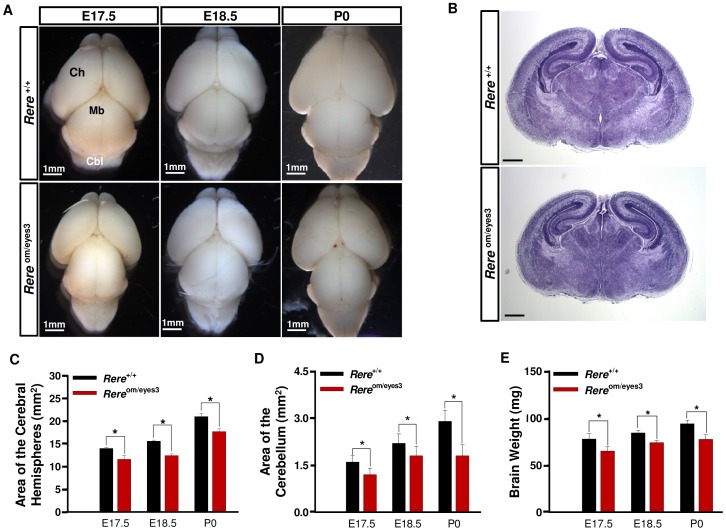
Brain hypoplasia in *Rere* ^om/eyes3^ embryos and mice. A) The brains of *Rere* ^om/eyes3^ embryos and mice appeared smaller than those of their wild-type litter mates at E17.5, E18.5 and P0 but no difference in overall morphology was observed between genotypes. B) Coronal brain sections from wild-type and *Rere* ^om/eyes3^ embryos at E18.5 were also comparable except for size. Representative examples of these sections are shown. Scale bar indicates 500 µm. C–D) The surface areas of the cerebral hemispheres (C) and cerebellum (D) were significantly reduced in *Rere* ^om/eyes3^ embryos and mice in comparison with those of wild-type embryos and mice between E17.5 and P0 (n≥5; * = *p*<0.03). E) Whole brain weights were also significantly decreased in *Rere* ^om/eyes3^ embryos and mice in comparison with those of wild-type embryos and mice between E17.5 and P0 (n≥5; * = p<0.01).

Since individuals with 1p36 deletions can have microcephaly and CNS anomalies and *Rere* ^om/om^ embryos have open neural tube defects, we examined *Rere* ^om/eyes3^ mice for evidence of abnormal brain development [1], [6], [7]. We specifically focused our attention on brain development from E17.5 to postnatal day 0 (P0) to avoid any secondary effects which could be caused by postnatal growth deficiency. Gross examination revealed that the overall brain morphology of the *Rere* ^om/eyes3^ embryos and mice was similar to that of their littermate controls at these ages (Fig. 3A). Histological analyses of the brain were also indistinguishable between *Rere* ^om/eyes3^ embryos and control embryos at E18.5 (Fig. 3B). However, the surface areas of the cerebral hemispheres and cerebellum were significantly reduced in the *Rere* ^om/eyes3^ embryos and newborn pups when compared to their wild-type littermates between E17.5 and P0 (Fig. 3A, 3C and 3D). The total brain weights of *Rere* ^om/eyes3^ embryos and newborn pups (P0) were also significantly decreased in comparison with wild-type embryos and mice (Fig. 3E). Since the body weight of *Rere* ^om/eyes3^ embryos and newborn pups were not statistically different from their wild-type littermates, these results suggest that RERE deficiency is associated with reduced brain size and brain weight independent of body weight.

### Decreased Number of the NeuN-positive Hippocampal Neurons in *Rere* ^om/eyes3^ Mice

**Figure 4:**
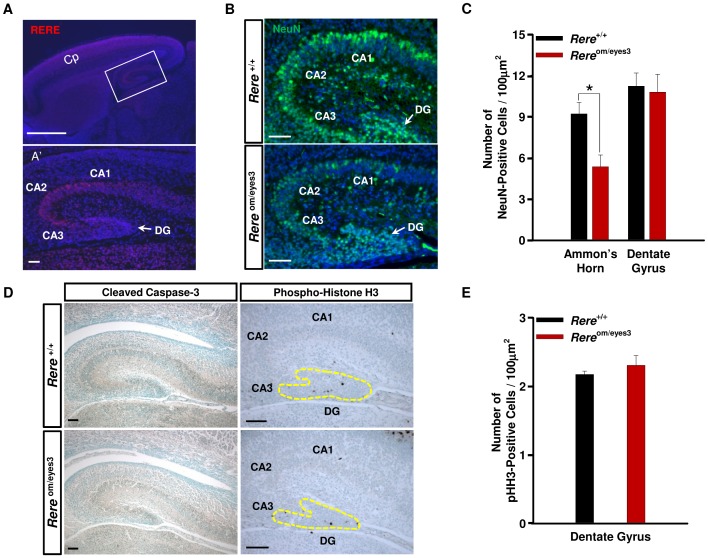
Decreased number of NeuN-positive neurons in the hippocampi of *Rere* ^om/eyes3^ embryos. A) RERE immunohistochemistry analyses of mid-sagittal sections of the brain at E18.5. Lower panel (A’) is higher magnification of the boxed area in the upper panel, which includes the hippocampus. RERE (red color) is primarily expressed in Ammon’s horn of the hippocampus at E18.5. Scale bar = 100 µm. B) Brain sections through the hippocampi of wild-type and *Rere* ^om/eyes3^ embryos were probed with anti-NeuN antibody (green color) at E18.5. Decreased numbers of NeuN-positive cells are seen in CA fields of the hippocampus from *Rere* ^om/eyes3^ embryos compared to those from wild-type embryos. Scale bar indicates 50 µm. C) NeuN-positive cells in Ammon’s horn, including CA1, CA2, and CA3, and the dentate gyrus were counted and normalized to the area of each region. The number of NeuN positive neurons per area was significantly decreased in the Ammon’s horns of *Rere* ^om/eyes3^ embryos when compared to wild-type embryos (analysis based on fifteen slides containing at least three sections for each of three or more embryos; * = *p*<0.01). D) Immunohistochemical analyses of brain section from E18.5 embryos probed using either an anti-cleaved Caspase-3 antibody and an anti-Phospho-Histone H3 (pHH3) antibody. Cleaved Caspase-3-positive cells were not detected in *Rere* ^om/eyes3^ embryos. Phospho-Histone H3-positive cells were identified in the dentate gyri (outlined with a dashed yellow line) of embryos of each genotype. Scale bar = 100 µm. E) Phospho-Histone H3-positive cells were counted and normalized to the area of the dentate gyrus including subgranularzone (analysis based on fifteen slides containing at least three sections for each of three or more embryos). The number of Phospho-Histone H3-positive hippocampal cells per µm^2^ was not significantly different between wild-type embryos and *Rere* ^om/eyes3^ embryos. CA = cornu ammonis; Cp = cortical plate; DG = dentate gyrus.

Cognitive impairment is the most common feature seen in patients with 1p36 deletions [1], [6], [7]. It is well established that the hippocampus plays a key role in learning and memory [28], [29]. Immunohistochemical analyses revealed that RERE is expressed in the mouse hippocampus at E18.5 (Fig. 4A). To determine if decreased RERE expression affected the hippocampus, we examined the hippocampi of *Rere* ^om/eyes3^ mice and wild-type littermates for cells expressing neuronal nuclear antigen (NeuN), a molecular marker of the latter stages of neuronal maturation [30]. Although NeuN-positive cells were detected in the hippocampi of *Rere* ^om/eyes3^ embryos at E18.5, the number of NeuN-positive cells appeared to be reduced compared to their wild-type littermates (Fig. 4B). When we quantified the number of NeuN-positive cells in Ammon’s horn, including the cornu ammonis (CA) fields, and normalized by area, the number of NeuN-positive neurons per µm^2^ was found to be significantly lower in *Rere* ^om/eyes3^ embryos when compared to their wild-type littermates (Fig. 4C). In contrast, the number of NeuN-positive cells in dentate gyrus per area was not changed (Fig. 4C).

To determine if the decrease in the number of NeuN- positive hippocampal neurons was due to increased levels of apoptosis, we performed immunohistochemical analyses for cleaved Caspase-3, a marker for apoptotic cells. However, no apoptotic cells were identified in the hippocampi of *Rere* ^om/eyes3^ embryos or their wild-type littermates at E18.5 (Fig. 4D).

Since the subgranular zone of dentate gyrus has neurogenic activity, we examined the dentate gyri of *Rere* ^om/eyes3^ embryos and wild-type littermates using a variety of antibodies including anti-Phospho-Histone H3 to determine mitotic activity, anti-Nestin and anti-GFAP to detect stem cells [31], [32] and anti-Calretinin to detect immature granule cells [33], [34]. In all cases, no difference was seen in the number of positive cells per area in the dentate gyri of *Rere* ^om/eyes3^ embryos and wild-type littermates at E18.5 (Fig. 4D–E, Fig. S3 and S4).

Although most of the granule cells in the hippocampus are located in the dentate gyrus, some can also be found in the CA3 field of Ammon’s horn 35. To determine if failure of granule cell maturation could contribute to the decreased level of NeuN-positive cells seen within the Ammon’s horns of *Rere* ^om/eyes3^ embryos, we compared the number of immature Calretinin-positive granule cells per area in the Ammon’s horns of *Rere* ^om/eyes3^ embryos and wild-type littermates at E18.5. However, we found no significant differences between the numbers of Calretinin-positive cells per area in the Ammon’s horns of embryos of these genotypes (Fig. S4).

### Neurobehavioral Testing In *Rere* ^+/om^ Mice

Neurobehavioral testing is a useful method for identifying abnormalities in CNS function in mice and can sometimes provide additional clues to role of genes in the brain. Since *Rere* ^eyes3/eyes3^ and *Rere* ^om/eyes3^ mice have adverse health problems that could impact the results of such testing, we performed a battery of neurobehavioral tests on *Rere* ^+/om^ mice, that have no known health issues, and wild-type control littermates on a pure C57BL/6J background. Specifically, locomotor activity and unconditioned anxiety were tested using an open field activity test, motor coordination, balance and skill learning were tested using a Rotarod test, sensorimotor gating was tested using a prepulse inhibition of acoustic startle test, and learning and memory were tested using a conditioned fear test. However, no significant differences were seen between *Rere* ^+/om^ mice and wild-type littermates in these tests (Figs. S5 and S6).

### Hearing Loss In *Rere* ^Om/eyes3^ Mice

**Figure 5:**
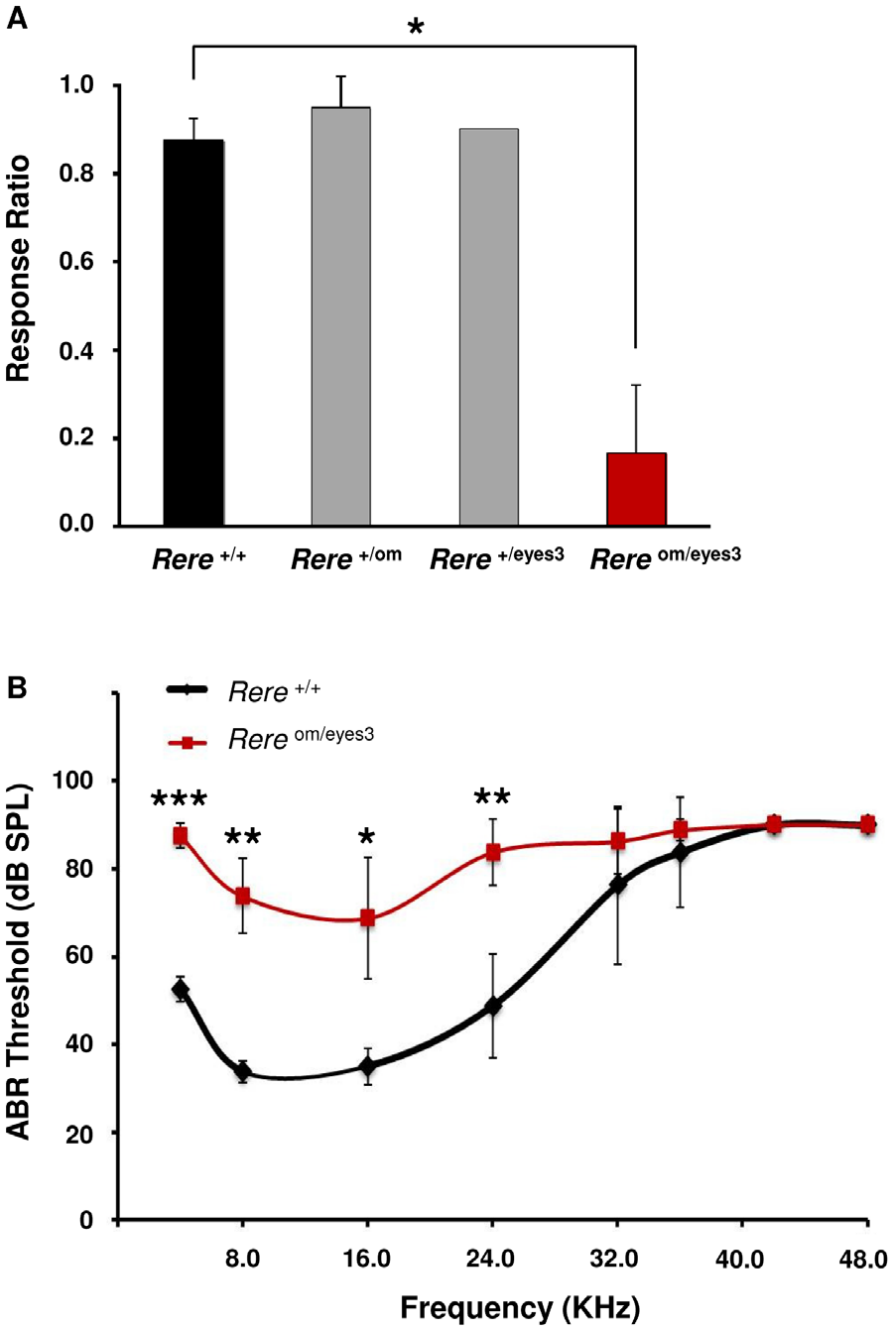
*Rere*om/eyes3 mice have reduced hearing potential compared to their wild-type littermates. A) The startle responses of *Rere* ^om/eyes3^ mice to a 108 dB sound burst at 19.9 kHz were significantly reduced compared to that of their wild-type littermates (*p* = 0.0003). Normal response patters were seen in *Rere* ^+/eyes3^ and *Rere* ^+/om^ mice. B) Auditory brainstem response (ABR) testing showed reduced hearing potential in the *Rere* ^om/eyes3^ mice compared to their wild-type littermates with an overall p-value <<0.001. The red line represents the ABR thresholds from *Rere* ^om/eyes3^ mice (n = 4) and the black line represents the ABR thresholds from wild-type littermates (n = 4; * = p<0.02, ** = p<0.005, *** = p<0.001).

Sensorineural hearing loss has been documented in children with both terminal and proximal interstitial 1p36 deletions 1,7. To determine if RERE deficiency can cause hearing loss in mice, we examined the startle responses of wild-type, *Rere* ^+/eyes3^, *Rere* ^+/om^ and *Rere* ^om/eyes3^ littermates at P21 to a 108 dB sound burst at 19.9 kHz emitted from a click box, a common screening test for severe hearing loss. While startle responses were similar for wild-type, *Rere* ^+/eyes3^ and *Rere* ^+/om^ mice, the *Rere* ^om/eyes3^ mice showed a significant decrease in their startle responses (Fig. 5A).

Auditory brainstem response (ABR) testing was conducted on *Rere* ^om/eyes3^ mice and their wild-type littermates at P21. *Rere* ^om/eyes3^ mice were found to have significantly elevated ABR thresholds between 4 kHz and 24 kHz when compared to their wild-type littermates (Fig. 5B).

### Cardiovascular Malformations In *Rere* ^Om/eyes3^ Embryos

Cardiovascular malformations and cardiomyopathy are commonly seen in both terminal and proximal 1p36 deletions 1,4,6,7. *Rere* ^om/om^ mice die between E9.5 and E11.5 with unlooped hearts and signs of cardiac failure suggesting that RERE may play a role in early cardiovascular development 18.

**Figure 6:**
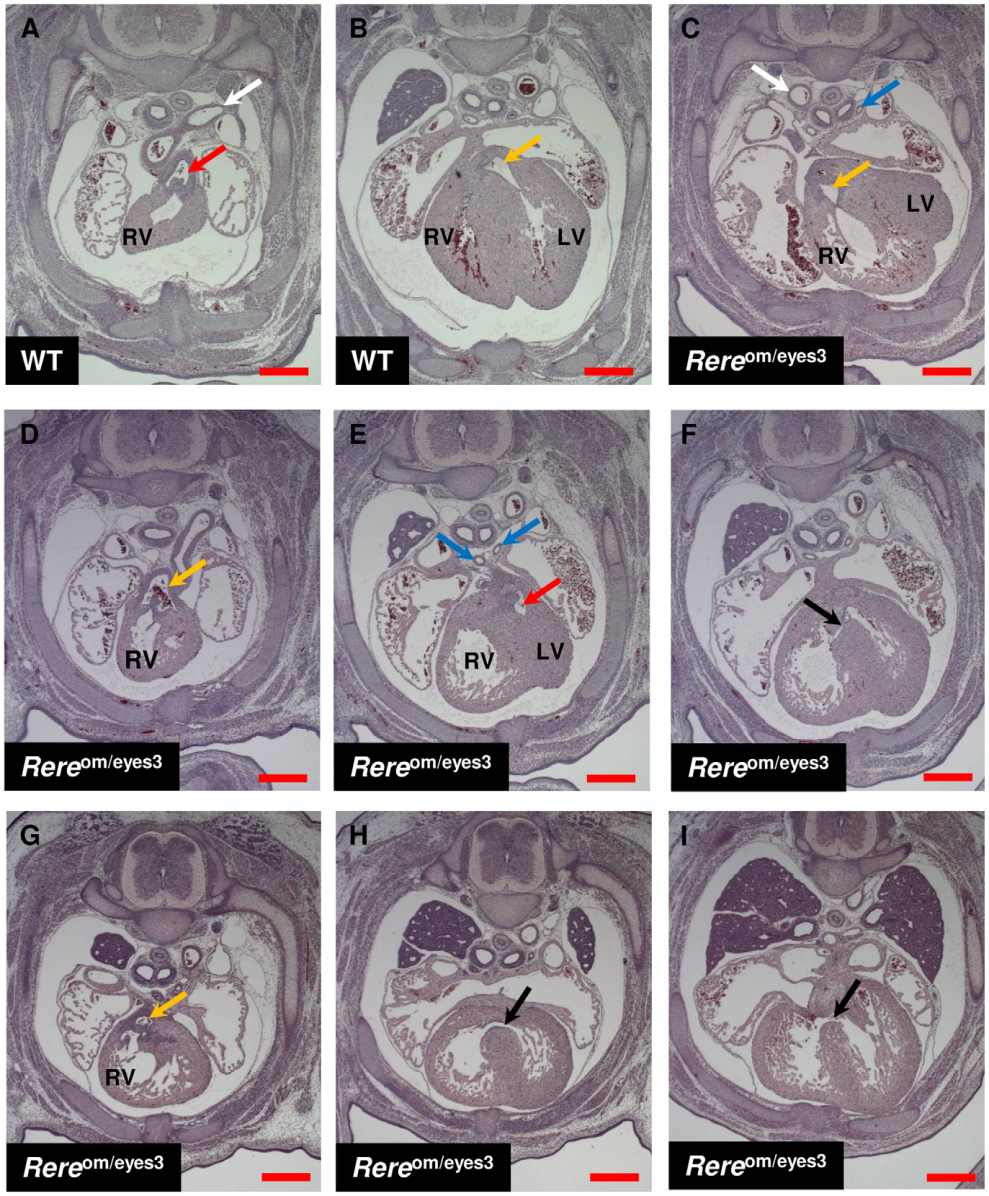
*Rere* ^om/eyes3^ embryos on a C57BL6background have cardiovascular malformations. Transverse sections of *Rere* ^om/eyes3^ embryos and wild-type littermate controls at E15.5. A–B) A wild-type embryo with a normal left-sided descending aorta (white arrow), a pulmonary artery that emerges from the right ventricle (red arrow), and an ascending aorta that emerges from the left ventricle (yellow arrow). C) An *Rere* ^om/eyes3^ embryo with a right-sided aorta (white arrow) with an aberrant left subclavian artery (not shown) and double outlet right ventricle in which ascending aorta emerges from the right ventricle (yellow arrow). In this embryo, the left pulmonary artery (blue arrow) originates abnormally from the ductus arteriosus. D–F) An *Rere* ^om/eyes3^ embryo with transposition of the great arteries in which the ascending aorta emerges from the right ventricle (yellow arrow) and the ductus arteriosus and left and right pulmonary arteries (blue arrows) emerge from the left ventricle. The red arrow in panel E points to the region of the left ventricle below the valve leading to the pulmonary artery. This embryo also has a periventricular septal defect (black arrow). G–I) An *Rere* ^om/eyes3^ embryo with double outlet right ventricle in which the ascending aorta (yellow arrow) emerges from the right ventricle. This embryo had two separate periventricular septal defects (black arrows in panel H and I). LV = Left ventricle, RV = right ventricle, red scale bars = 0.5 mm.

To determine if RERE deficiency leads to the development of cardiovascular malformations in the latter stages of embryonic heart development, we examined H&E-stained transverse sections from *Rere* ^om/eyes3^ embryos harvested at E15.5 from breedings between *Rere* ^+/om^ and *Rere* ^+/eyes3^ mice. On a mixed B6Brd/129S6 background, we identified no cardiovascular malformations in the *Rere* ^om/eyes3^ embryos examined (n = 3). However, since cardiovascular phenotypes are often strain dependent, we repeated these studies using breeding pairs of *Rere* ^+/om^ mice on a pure C57BL/6 background and *Rere* ^+/eyes3^ mice that had been backcrossed for at least 6 generations onto a C57BL/6J background. On this background, all of the *Rere* ^om/eyes3^ embryos (6/6, 100%) had one or more cardiovascular malformations that were not seen in wild-type littermate controls (n = 3). These results are summarized in Table 2 and representative transverse sections from these embryos are show in Figure 6.

**Table 2:** Cardiovascular malformations are seen in *Rere* ^om/eyes3^ embryos on a C57BL6 background.

Genotype	Embryo ID	Cardiovascular malformations
*Rere* ^om/eyes3^	1	Right-sided aortic arch with aberrant left subclavian artery, ventricular septal defect
*Rere* ^om/eyes3^	2	Right-sided aortic arch with aberrant left subclavian artery, aberrant left pulmonary artery arising from the left common carotid artery, double outlet right ventricle
*Rere* ^om/eyes3^	3	Right-sided aortic arch with aberrant left subclavian artery, aberrant left pulmonary artery arising from the ductus arteriosus, double outlet right ventricle, ventricular septal defect
*Rere* ^om/eyes3^	4	Left-sided aortic arch with aberrant right subclavian artery
*Rere* ^om/eyes3^	5	Left-sided aortic arch, double outlet right ventricle, ventricular septal defect X 2
*Rere* ^om/eyes3^	6	Left-sided aortic arch, transposition of the great arteries, ventricular septal defect
Wild-type	1–3	None

doi:10.1371/journal.pone.0057460.t002

Aortic arch anomalies were seen in 60% (4/6) of *Rere* ^om/eyes3^ embryos. Three embryos had right-sided aortic arches with aberrant left subclavian arteries (Fig. 6C). Two of these embryos also had abnormal left pulmonary arteries. In one embryo the left pulmonary artery arose from the ductus arteriosus (Fig. 6C) and in the other the left pulmonary artery arose from the left common carotid artery. A fourth *Rere* ^om/eyes3^ embryo had a left-sided aortic arch with an aberrant right subclavian artery.

Double outlet right ventricle–a congenital heart defect in which the aorta arises from the right ventricle–was seen in 50% (3/6) of *Rere* ^om/eyes3^ embryos (Fig. 6C and 6G). One embryo with a left-sided aortic arch was found to have transposition of the great arteries (1/6, 17%) in which the aorta arises from the right ventricle and the pulmonary artery arises from the left ventricle (Fig. 6D and 6E). Two of the embryos with double outlet right ventricle and the embryo with transposition of the great arteries also had clear evidence of one or more perimembranous ventricular septal defects (Fig 6F, 6H and 6I). A perimembranous ventricular septal defect was also seen in one embryo with a right-sided aortic arch with aberrant left subclavian artery but no evidence of a conotruncal abnormality.

### Spontaneous Development Of Cardiac Fibrosis In *Rere* ^Om/eyes3^ Adult Mice

**Figure 7:**
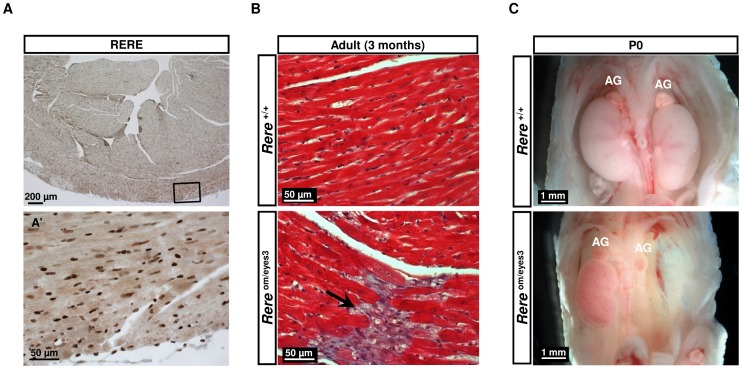
Cardiac fibrosis and renal agenesis in *Rere* ^om/eyes3^ mice. A) RERE nuclear staining was detected in the endocardium, myocardium, and epicardium in cardiac sections from 4 month-old wild-type mice. Lower panel (A’) is higher magnification of a boxed area in upper panel. B) Cardiac sections from 3 month-old wild-type (upper panel) and *Rere* ^om/eyes3^ (lower panel) mice. Masson’s trichrome staining revealed areas of interstitial fibrosis (black arrow) in sections from the *Rere* ^om/eyes3^ mouse that were not seen in those from the wild-type mouse. C) Renal agenesis in an *Rere* ^om/eyes3^ mouse at P0 with normal development of the adrenal gland (AG).

To determine if RERE deficiency could have adverse effects on the postnatal heart, we first used immunohistochemistry to confirm that RERE was expressed in the adult heart. At 4 months of age, we found that RERE is expressed in the endocardium, myocardium and epicardium (Fig. 7A). Next, we examined the hearts of a group (n = 3) of *Rere* ^om/eyes3^ adult mice between 16 months and 20 months of age and age matched controls. Trichrome staining of cardiac sections from two of the *Rere* ^om/eyes3^ mice (2/3, 66%) revealed areas of fibrosis in the myocardium of the ventricle wall. Similar areas of fibrosis were not seen in sections from the other *Rere* ^om/eyes3^ mouse or controls. We then repeated these studies with a second group of *Rere* ^om/eyes3^ mice (n = 4) between 3 and 5 months of age. Multiple areas of fibrosis were seen in trichrome-stained cardiac sections from one 3-month old *Rere* ^om/eyes3^ mouse (1/4, 25%) but not in cardiac sections from the other *Rere* ^om/eyes3^ mice or controls (Fig. 7B).

### Renal Agenesis In *Rere* ^Eyes3/eyes3^ And *Rere* ^Om/eyes3^ Mice

Unilateral renal agenesis is occasionally seen in *Rere* ^eyes3/eyes3^ mice but is more commonly seen in *Rere* ^om/eyes3^ mice. Approximately 37% (13/35) of *Rere* ^om/eyes3^ mice evaluated at P0 showed evidence of renal agenesis. In 77% of cases (10/13) the agenesis was bilateral and in 23% (3/13) of cases it was unilateral. In all cases the adrenal glands were unaffected (Fig. 7C).

## Discussion

### Rere-Deficient Mice Reveal The Potential Contribution Of *Rere* To The Development Of 1p36 Deletion Phenotypes

Most symptomatic terminal and interstitial deletions of chromosome 1p36 involve multiple genes. This makes it difficult to identify individual genes that contribute to specific 1p36 deletion phenotypes using human cytogenetic data alone. While it is possible that the expanded use of high-resolution copy number detection techniques and whole exome or whole genome sequencing will ultimately provide clear evidence for the role of some 1p36 genes–particularly those with strong effects–mouse models can provide an effective alternative means of identifying the dosage-sensitive genes that are likely to contribute to specific 1p36 phenotypes.

*RERE* is located in the proximal region of chromosome 1p36. Due to its role as a nuclear receptor coregulator and the role it plays in retinoic acid signaling, we identified it as an attractive candidate gene which could contribute to the development of several phenotypes seen in individuals with proximal interstitial deletions or large terminal deletions of 1p36 2,6–8,18–20. Using an allelic series of mice, we have shown that RERE deficiency leads to a spectrum of defects that includes microphthalmia, postnatal growth deficiency, brain hypoplasia, decreased numbers of NeuN-positive hippocampal neurons, hearing loss, cardiovascular malformations, spontaneous development of cardiac fibrosis and renal agenesis (Table 3). These findings suggest that RERE plays a critical role in the development and function of multiple organs– including the eye, brain, inner ear, heart and kidney–that are also affected in individuals with terminal and proximal interstitial deletions of 1p36 that include *RERE* (Table 3) 1,4,6,7. This suggests that deletions of *RERE* may contribute–alone or in conjunction with other genetic, environmental, or stochastic factors–to the development of many of the phenotypes seen in individuals with terminal and interstitial deletions that include the proximal region of chromosome 1p36.

**Table 3:** Summary of phenotypes seen in 1p36 deletion patients and RERE-deficient mice and embryos.

Organ	Terminal 1p36 deletions†	Proximal 1p36 interstitial deletions††	*Rere* ^eyes3/eyes3^	*Rere* ^om/eyes3^	*Rere* ^om/om^ *
Eye	Eye and vision problems	Eye and vision problems	Micro-phthalmia	Micro-phthalmia	Fusion of the optic and telen-cephalic vesicles
Somatic growth	Postnatal growth deficiency	Postnatal growth deficiency	Small size	Postnatal growth deficiency	N/A
CNS	Intellectual disability, seizures, behavioral problems,structural defects	Intellectual disability, seizures, behavioral problems, structural defects	–	Brain hypoplasia, decreased number of NeuN+ hippocampal cells	Open neural tube defects
Inner ear	Hearing loss	Hearing loss	–	Hearing loss	N/A
Kidney	Renal anomalies	–	Renal agenesis	Renal agenesis	N/A
Heart	Cardiovascular malformations, cardiomyopathy	Cardiovascular malformations, cardiomyopathy	–	Cardiovascular malformations, cardiac fibrosis	Failure of cardiac looping, cardiac failure

N/A = not applicable due to early embryonic lethality;

– = phenotypes in this organ system have not be documented;

† = References 1,4,

†† = References 6,7;

* = Reference 18.

doi:10.1371/journal.pone.0057460.t003

Although *Rere* ^+/eyes3^ and *Rere* ^+/om^ mice have no apparent phenotypes on a mixed B6Brd/129S6 background, it would be premature to conclude that haploinsufficiency of *RERE* alone is insufficient to cause phenotypes in humans. There are clear examples in which phenotypes caused by haploinsufficiency in humans are not recapitulated in haploinsufficient mice but are seen in mice bearing recessive mutations in the same gene 36,37. It is also possible that *Rere* ^+/eyes3^ or *Rere* ^+/om^ mice would have phenotypes on a different strain background 38–41. Indeed, the effect of strain background is clearly seen in the fact that no cardiovascular malformations were identified in *Rere* ^om/eyes3^ mice on a mixed B6Brd/129S6 background but were clearly present on a C57BL6 background.

Since multiple genes are deleted in both terminal and interstitial 1p36 deletions, it is also possible that haploinsufficiency of *RERE* contributes to the development of specific phenotypes in conjunction with another gene or genes within deleted intervals–with haploinsufficiency of each gene adding to the total genetic load and increasing the likelihood that the individual will develop clinical symptoms. The likelihood of developing clinical symptoms may be further modified by variations in the expression and function of genes outside of the 1p36 region as well as environmental or stochastic factors. Indeed, the influence of such factors is clearly seen in the incomplete penetrance and variable expressivity evident in *Rere* ^eyes3/eyes3^ mice some of which appear normal while others are clearly affected by microphthalmia and renal agenesis.

### The Potential Effects Of Decreased Retinoic Acid Signaling In RERE-Deficient Mice

Vilhais-Neto and colleagues have shown that RERE is a positive regulator of retinoic acid signaling–both *in vitro* and *in vivo*–and that decreased retinoic acid signaling activity leads to abnormal somite development in *Rere* ^om/om^ embryos 20. In addition to its role in somitogenesis, retinoic acid signaling is required for normal development of the eye, brain, inner ear, heart and kidney 42. We have shown that *Rere* ^om/eyes3^ embryos and mice have defects in each of these organs. It seems plausible, therefore, that dysregulation of retinoic acid signaling will ultimately be shown to contribute to the development of many of the defects seen in RERE-deficient mice. The potential role of decreased retinoic acid signaling in each of the phenotypes seen in RERE-deficient mice is discussed in greater detail in the sections that follow.

### The Role Of RERE In Eye Development

The first evidence of RERE’s role in murine eye development came from Zoltewicz and colleagues who showed that the optic and telencephalic vesicles of *Rere* ^om/om^ embryos are fused making the optic vesicles appear morphologically absent at E9.5 18. In this report we have shown that *Rere* ^eyes3/eyes3^ and *Rere* ^om/eyes3^ mice have unilateral or bilateral microphthalmia, providing additional evidence of this role. It is interesting to note that small eyes are also associated with truncating recessive mutations in *rerea*, the zebrafish homologue of *RERE* 43,44.

In mice, retinoic acid signaling has been shown to be required for reciprocal interactions between the optic vesicle and the invaginating lens placode and promotes normal development of the ventral retina and optic nerve through its activities in the periocular mesenchyme 45. In humans, abnormalities in retinoic acid signaling have also been seen in association with microphthalmia. For example, autosomal recessive mutations in the *STRA6* gene–which encodes a high-affinity cell-surface receptor for retinol/retinol binding protein complexes that mediates the cellular uptake of retinol into the cell–cause syndromic microphthalmia as part of Matthew-Wood syndrome (microphthalmia, syndromic 9; OMIM **#**601186) 46.

To date, eye anomalies have not been described in individuals with proximal interstitial deletions of 1p36 6,7. This suggests that haploinsufficiency of *RERE*, by itself, is not sufficient to cause severe eye defects in humans. We cannot rule out the possibility that *RERE* contributes–along with other factors–to the eye defects seen in individuals with large terminal deletions. However, the eye defects associated with terminal 1p36 deletions are typically less severe than those seen in *Rere* ^eyes3/eyes3^ and *Rere* ^om/eyes3^ mice and include strabismus, refractive errors, nystagmus, cataracts, retinal albinism and colobomas 1,4.

### The Role Of RERE In Postnatal Growth Deficiency

Postnatal growth deficiency is a common feature of individuals with both terminal and proximal interstitial deletions of 1p36 4,6,7. This phenotype is also seen in RERE-deficient mice with *Rere* ^om/eyes3^ mice having normal birth weights but showing growth deficiency by 1 week of age and a plateau in growth at 6 weeks of age. Small size was also noted in *Rere* ^eyes3/eyes3^ mice.

At the present time it is unclear whether growth deficiency is a primary effect of RERE deficiency or if it is secondary to other phenotypes seen in these mice. However, since RERE is known to interact with the nuclear receptor TLX, it is interesting to note that *Tlx*-null mice have a similar pattern of postnatal growth deficiency 19,47. Specifically, *Tlx*-null mice are the same size as their wild-type littermates at birth but show evidence of growth deficiency over the first three weeks of life 47. Future studies will be needed to determine the mechanism by which RERE affects somatic growth and whether RERE interacts with TLX in this process.

### The Role Of RERE In Brain Development

A variety of CNS abnormalities have been described in patients with both terminal and proximal interstitial deletions of 1p36 including enlargement of the ventricles, cortical atrophy, leukoencephalopathy, agenesis or abnormalities of the corpus callosum and septum pellucidum, and polymicrogyria 1,4,6,7. Microcephaly–or small head size–is also commonly seen in individuals with 1p36 deletions and is typically caused by a failure of normal brain growth 1,4,6,7,48,49. In this study, structural brain anomalies were not identified in *Rere* ^om/eyes3^ embryos, but the cerebrum and cerebellum of these embryos were significantly smaller than those of their wild-type littermates. This reduction in brain size was independent of somatic growth deficiency. This suggests that RERE deficiency may contribute to reduced brain size and the development of microcephaly in individuals with 1p36 deletions.

Defects in cognitive function are the most prevalent phenotype reported in terminal and proximal interstitial deletions of 1p36 1,3,4,6. The hippocampus has been established to be a critical region for learning and memory and abnormalities in the hippocampus, or its neurons, are seen in various mouse models that display cognitive defects 28,29. We have shown that RERE is expressed in the hippocampus and that the number of NeuN-positive hippocampal neurons per µm^2^ was significantly decreased in the Ammon’s horns of *Rere* ^om/eyes3^ embryos at E18.5. This reduction did not appear to be due to increased levels of apoptotic cell death within this region.

Since expression of NeuN is considered a good marker of the latter stages of neuronal maturation, we considered the possibility that the decrease in NeuN-positive neurons in Ammon’s horn could be due, in part, to failure of proper differentiation of granule cells in the CA3 field. However, we found that the number of immature Calretinin-positive granule cell neurons per area in the Ammon’s horns of *Rere* ^om/eyes3^ and wild-type embryos to be comparable. Additional studies are underway to determine if impaired neurogenesis, including differentiation of pyramidal cells in the CA fields, contributes to the decreased numbers of NeuN-positive neurons seen in the Ammon’s horns of *Rere* ^om/eyes3^ embryos.

In contrast to our findings in Ammon’s horn, the numbers of NeuN-positive cells per area in the dentate gyrus were not significantly different between *Rere* ^om/eyes3^ embryos and wild-type littermates. Further analyses revealed no difference in the mitotic activity, the number of Nestin-positive or GFAP-positive stem cells or in the number of immature granule cells in the dentate gyri of embryos of these genotypes.

Neurobehavioral testing was performed on *Rere* ^+/om^ mice since they do not have the health problems seen in *Rere* ^om/eyes3^ and *Rere* ^eyes3/eyes3^ mice which could compromise results. However, we found no significant differences between *Rere* ^+/om^ and wild-type mice in open field activity, Rotarod, prepulse inhibition of acoustic startle and conditioned fear tests. This result was not completely unexpected since it is likely that multiple genes on chromosome 1p36 contribute to cognitive defects 9–12. The generation of *Rere* conditional knockout mice would enhance our ability to gauge the potential contribution of RERE deficiency on cognitive ability and behavior by allowing us to decrease the expression of RERE in specific brain areas–like the hippocampus–while avoiding deleterious systemic effects that could compromise test results.

### The Role Of RERE In The Auditory System

Sensorineural hearing loss is seen in some individuals with terminal and interstitial deletions of 1p36 1,7. *Rere* ^om/eyes3^ mice performed poorly on both acoustic startle and ABR testing. This suggests that RERE is required for normal hearing in mice. Similar findings have been seen in zebrafish in which autosomal recessive mutations in the *RERE* homolog, *rerea*, cause inconsistent startle response and decreased microphonic potentials 50. Studies are underway to determine the expression pattern of RERE in the inner ear and the molecular mechanism by which RERE deficiency affects inner ear development and/or function.

### The Role Of RERE In The Heart

Cardiovascular malformations are common findings in both terminal and proximal interstitial deletions of 1p36 1,4,6,7. *Rere* ^om/om^ embryos have been shown to die around E9.5–E11.5 with unlooped hearts and signs of cardiac failure 18. This suggests that RERE plays a critical role in early cardiovascular development. Failure of cardiac looping is also seen in embryos in which both copies of *Raldh2*–a gene which encodes the enzyme that catalyzes the production of retinoic acid in most tissues–have been disrupted 51,52. Given the similarities seen in RERE and RALDH2-deficient mice, and the fact that RERE has been established to be a positive regulator of retinoic acid signaling, it is likely that RERE functions to regulate retinoic acid signaling in early cardiovascular development 20.

In contrast to the early embryonic lethality seen in *Rere* ^om/om^ embryos, *Rere* ^om/eyes3^ embryos can be readily obtained at E15.5 but have a variety of cardiovascular malformations that include aortic arch anomalies, double outlet right ventricle, transposition of the great arteries and perimembranous ventricular septal defects.

Aortic arch anomalies arise from errors in the embryological development of the branchial arches 53. The most common aortic arch anomaly in *Rere* ^om/eyes3^ embryos, was right-sided aortic arch with aberrant left subclavian artery. This defect occurred in 50% of *Rere* ^om/eyes3^ embryos. Left-sided aortic arch with aberrant right subclavian artery was also observed. Aortic arch anomalies are not commonly associated with 1p36 deletions but have been reported 32.

In the developing heart, proper connection of the pulmonary artery to the right ventricle and the aorta to the left ventricle requires the fusion and spiraling of two opposing ridges of endocardial cushion tissue to form the conotruncal septum 54,55. Failure of the conotruncal septum to spiral results in transposition of the great arteries with the pulmonary artery being connected to the left ventricle and the aorta being connected to the right ventricle. Misalignment of the conotruncus can result in double outlet right ventricle, where the aorta and the pulmonary artery connect to the right ventricle. These conotruncal defects– transposition of the great arteries or double outlet right ventricle–were seen in 66% of *Rere* ^om/eyes3^ embryos. Although these particular conotruncal defects are not commonly reported in individuals with 1p36 deletions, tetralogy of Fallot–a related conotruncal anomaly consisting of an overriding aorta, ventricular septal defect, pulmonary valve stenosis and hypertrophy of the right ventricle–is among the most common cardiovascular defects seen in individuals with 1p36 deletions 1,4.

Failure of the conotruncus to connect to the muscular interventricular septum also results in a perimembranous ventricular septal defect 54. Perimembranous ventricular septal defects were clearly seen in 66% of *Rere* ^om/eyes3^ embryos including one embryo without evidence of a conotruncal defect. Ventricular septal defects are seen in approximately 23% of patients with terminal deletions of 1p36 and have also been seen in patients with interstitial deletions involving *RERE* 1,6.

A similar pattern of aortic arch anomalies, conotruncal defects and ventricular septal defects are seen in the progeny of vitamin A-deficient rat dams, retinoic X receptor alpha-null (RXRα^−/−^) mice and mice that are deficient for various combinations of retinoic acid receptors (RARs) 33,35,56,57. Given RERE’s known role as a positive regulator of retinoic acid signaling, it is likely that the cardiovascular malformations seen in *Rere* ^om/eyes3^ embryos are due, at least in part, to abnormal retinoic acid signaling in the developing heart 20.

Retinoic acid is also known to suppress cardiac fibrosis and myocardial hypertrophy 58,59. This may help to explain why a portion of *Rere* ^om/eyes3^ mice on a mixed B6Brd/129S6 background spontaneously develop cardiac fibrosis. Although RERE is widely expressed in adult heart, it is unclear whether the development of fibrosis is due to the cell autonomous role of RERE in cardiomyocytes or if the fibrosis seen in *Rere* ^om/eyes3^ mice is secondary to RERE’s effects on other organ systems. Differentiating between these two possibilities will require the generation and testing of cardiomyocyte-specific *Rere* conditional knockout mice.

### The Role Of RERE In Kidney Development

Renal anomalies are seen in about 22% of individuals with 1p36 deletions 1. Typically these defects are less severe than the unilateral and bilateral kidney agenesis seen in the *Rere* ^eyes3/eyes3^ and Rere^om/eyes3^ mice. Although one individual with a 1p36 deletion and a solitary kidney has been reported by the Unique - Rare Chromosome Disorder Support Group (www.rarechromo.org), the extent of this patient’s deletion was not described so it is unclear whether *RERE* was deleted.

Bilateral renal agenesis is incompatible with life and contributes to the high level of perinatal mortality seen in *Rere* ^om/eyes3^ mice. Renal anomalies are seen in a variety of mouse models with defects in retinoic acid signaling with renal agenesis being specifically described in double heterozygous RARα^+/−^;RARγ^+/−^ embryos 60. This suggests that RERE deficiency may cause renal agenesis in mice through disruption of retinoic acid signaling.

## Supporting Information

Figure S1 ***Rere*^eyes3/eyes3^ mice have decreased body weight compared to their wild-type littermates.** *Rere* ^eyes3/eyes3^ and wild-type mice were weighed at 3, 7 and 10 weeks of age (n = 5–8). At all time points the *Rere* ^eyes3/eyes3^ mice weighed significantly less than their wild-type littermates. * = *p*<0.001.
(TIF)

Figure S2 **The expression level of the RERE protein is not affected by the** ***eyes3*** **mutation.** Quantification of western blot analyses demonstrates that the level of RERE protein, normalized to the level of β-actin, is not significantly different between wild-type, *Rere* ^eyes3/eyes3^ and *Rere* ^+/eyes3^ embryos at E10.5.
(TIF)

Figure S3 **The number of Nestin-positive and GFAP-positive cells per area in the dentate gyrus of the hippocampi of** ***Rere*** **^om/eyes3^ and wild-type embryos is not significantly different.** Brain sections from *Rere* ^om/eyes3^ and wild-type embryos were probed with anti-Nestin antibodies (A) or anti-GFAP antibodies (B). At E18.5, Nestin-positive cells are detected between the dentate granular cell layer (GCL) and the subgranular zone (SGZ). GFAP-positive cells are also found in a similar region of dentate gyrus. Red arrows indicate Nestin-positive cells. Red arrow heads point to GFAP-positive cells. Scale bar = 25 µm. C) Nestin-positive cells in dentate gyrus were counted and normalized to the area of dentate gyrus. The number of Nestin-positive cells per area was not significantly different between *Rere* ^om/eyes3^ embryos and wild-type embryos (analysis based on fifteen slides containing at least three sections for each of three or more embryos). D) GFAP-positive cells were quantified in the dentate gyrus and normalized to the area of dentate gyrus. The number of GFAP-positive cells per area was not significantly different between *Rere* ^om/eyes3^ embryos and wild-type embryos (analysis based on fifteen slides containing at least three sections for each of three or more embryos). GCL = granule cell layer; SGZ = subgranular zone.
(TIF)

Figure S4 **Number of calretinin-positive neurons per area is not changed in the Ammon’s horns or the dentate gyri of** ***Rere*** **^om/eyes3^ embryos compared to wild-type embryos.** A) Coronal sections showing the hippocampus were prepared from wild-type and *Rere* ^om/eyes3^ embryos and probed with anti-Calretinin antibodies at E18.5. Calretinin-positive cells are abundant in the dentate gyrus. In contrast, a few Calretinin-positive cells are detected in CA3 field. Scale bar indicates 100 µm. B–C) Calretinin-positive cells in the Ammon’s horns, including CA1, CA2, and CA3, and in the dentate gryi of embryos of each genotype were counted and normalized to the area of each region. Number of Calretinin-positive cells per area is not significantly different between the Ammon’s Horns or the dentate gyri of *Rere* ^om/eyes3^ embryos in comparison with their wild-type littermates (analysis based on twenty slides containing at least three sections for each of three or more embryos).
(TIF)

Figure S5 **Neurobehavioral analyses do not show a significant difference between wild-type and** ***Rere*** **^+/om^ littermates.** A–B) In open field activity testing, no difference was seen in the total distance traveled (panel A; *p* = 0.247) or the center distance:total distance ratio (panel B; *p* = 0.832) between wild-type and *Rere* ^+/om^ mice. C–D) In the Rotarod test, no difference was seen between wild-type and *Rere* ^+/om^ mice in the average time spent on the rod (panel C; *p* = 0.444) or the learning index (panel D; *p* = 0.054). No differences were seen between *Rere* ^+/om^ mice and their wild-type littermates in tests of acoustic startle (panel E; arbitrary units, *p* = 0.832) and prepulse inhibition (panel F; *p* = 0.176). Between 12 and 18 mice per genotype were used for these tests.
(TIF)

Figure S6 **Conditioned fear testing showed no differences between wild-type and** ***Rere*** **^om/+^ littermates.** A) The percent freezing on the training day (day 1) was not found to be significantly different between wild-type and *Rere* ^+/om^ mice (n = 12–18 per genotype). B) The average percent freezing to contextual clues was not different between genotypes (*p* = 0.469). C) The average percent freezing with the conditioned stimulus minus the percent freezing with the pre-conditioned stimulus (CS - preCS) was not different between genotypes (*p* = 0.953).
(TIF)
