# Supplementary figures and images for: An Allelic Series of Mice Reveals a Role for RERE in the Development of Multiple Organs Affected in Chromosome 1p36 Deletions
Source: PLoS One. 2013 Feb 25;8(2):e57460. doi: 10.1371/journal.pone.0057460 (PMC3581587; doi:10.1371/journal.pone.0057460)

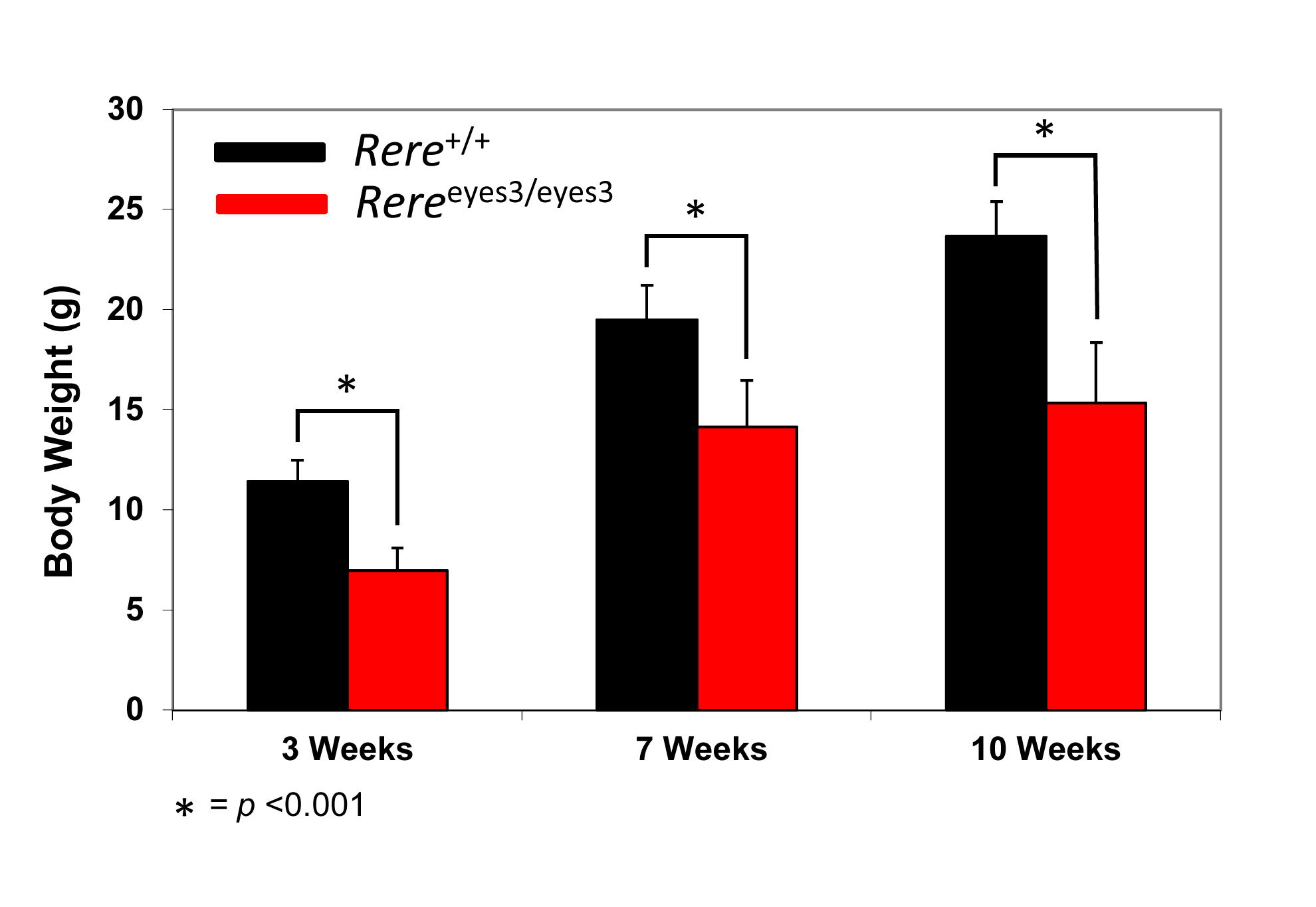

Supplement: Figure S1 — Rereeyes3/eyes3 mice have decreased body weight compared to their wild-type littermates. Rere eyes3/eyes3 and wild-type mice were weighed at 3, 7 and 10 weeks of age (n = 5–8). At all time points the Rere eyes3/eyes3 mice weighed significantly less than their wild-type littermates. * = p<0.001. (TIF) [file pone.0057460.s001.tif]

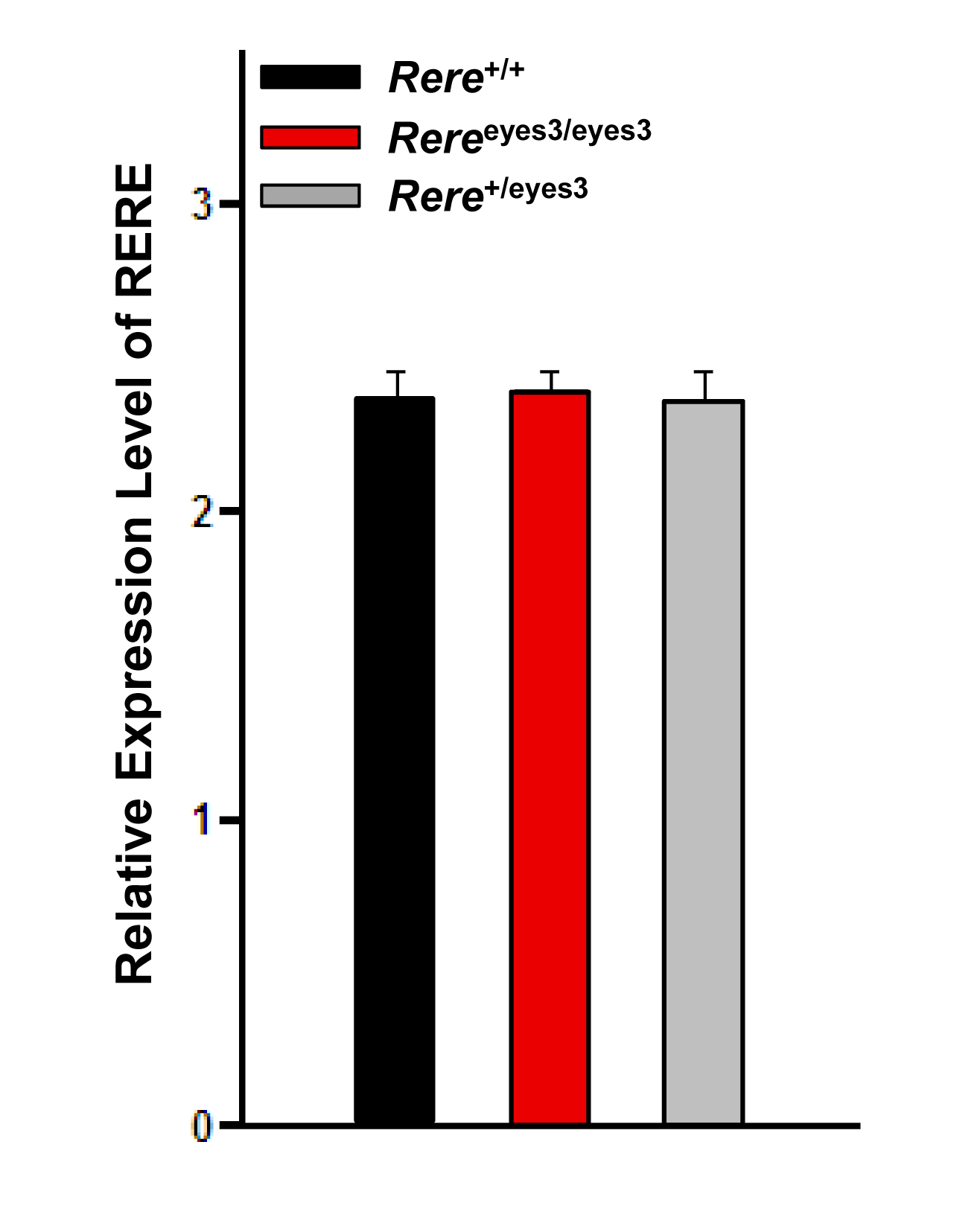

Supplement: Figure S2 — The expression level of the RERE protein is not affected by the eyes3 mutation. Quantification of western blot analyses demonstrates that the level of RERE protein, normalized to the level of β-actin, is not significantly different between wild-type, Rere eyes3/eyes3 and Rere +/eyes3 embryos at E10.5. (TIF) [file pone.0057460.s002.tif]

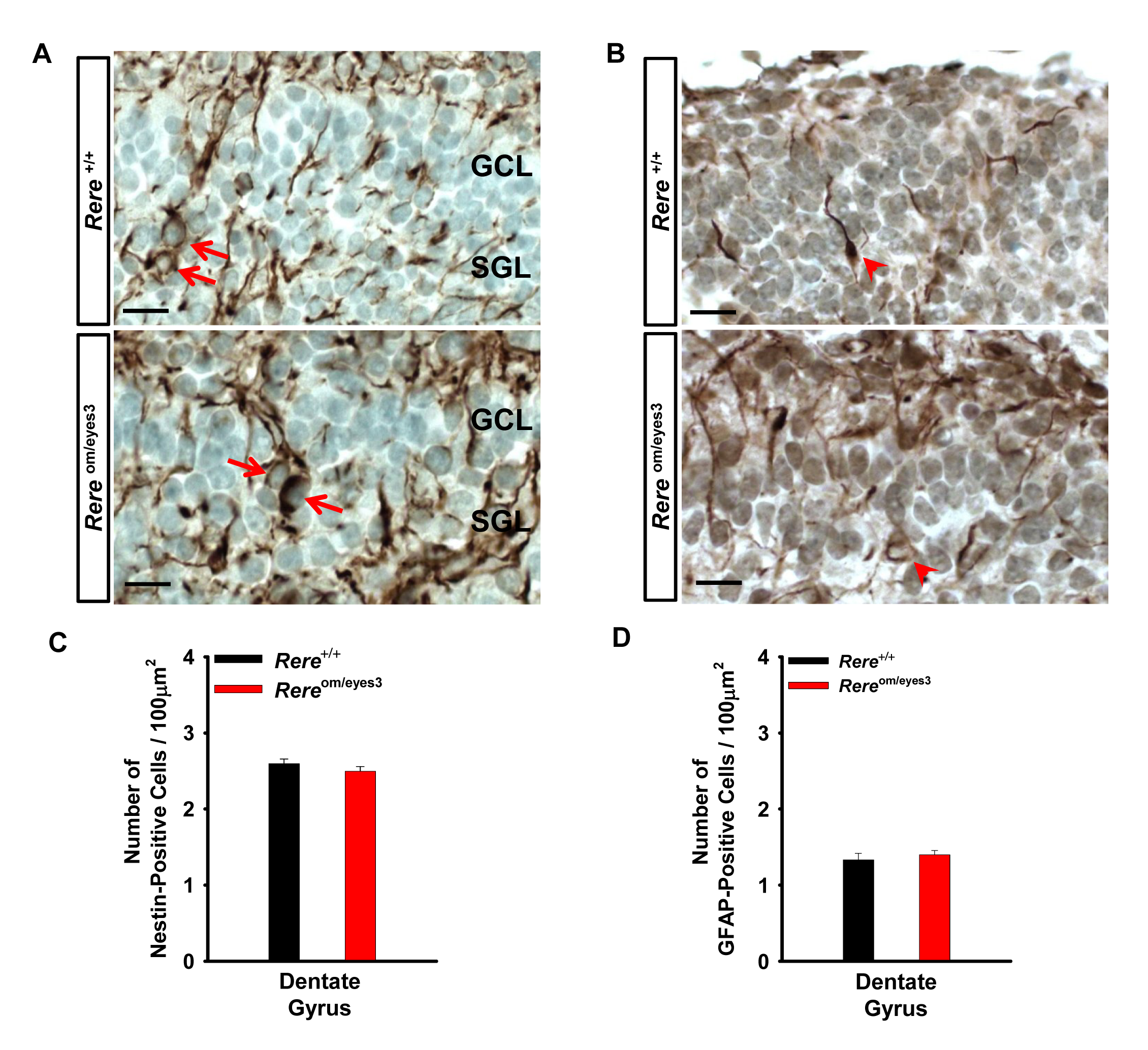

Supplement: Figure S3 — The number of Nestin-positive and GFAP-positive cells per area in the dentate gyrus of the hippocampi of Rere om/eyes3 and wild-type embryos is not significantly different. Brain sections from Rere om/eyes3 and wild-type embryos were probed with anti-Nestin antibodies (A) or anti-GFAP antibodies (B). At E18.5, Nestin-positive cells are detected between the dentate granular cell layer (GCL) and the subgranular zone (SGZ). GFAP-positive cells are also found in a similar region of dentate gyrus. Red arrows indicate Nestin-positive cells. Red arrow heads point to GFAP-positive cells. Scale bar = 25 µm. C) Nestin-positive cells in dentate gyrus were counted and normalized to the area of dentate gyrus. The number of Nestin-positive cells per area was not significantly different between Rere om/eyes3 embryos and wild-type embryos (analysis based on fifteen slides containing at least three sections for each of three or more embryos). D) GFAP-positive cells were quantified in the dentate gyrus and normalized to the area of dentate gyrus. The number of GFAP-positive cells per area was not significantly different between Rere om/eyes3 embryos and wild-type embryos (analysis based on fifteen slides containing at least three sections for each of three or more embryos). GCL = granule cell layer; SGZ = subgranular zone. (TIF) [file pone.0057460.s003.tif]

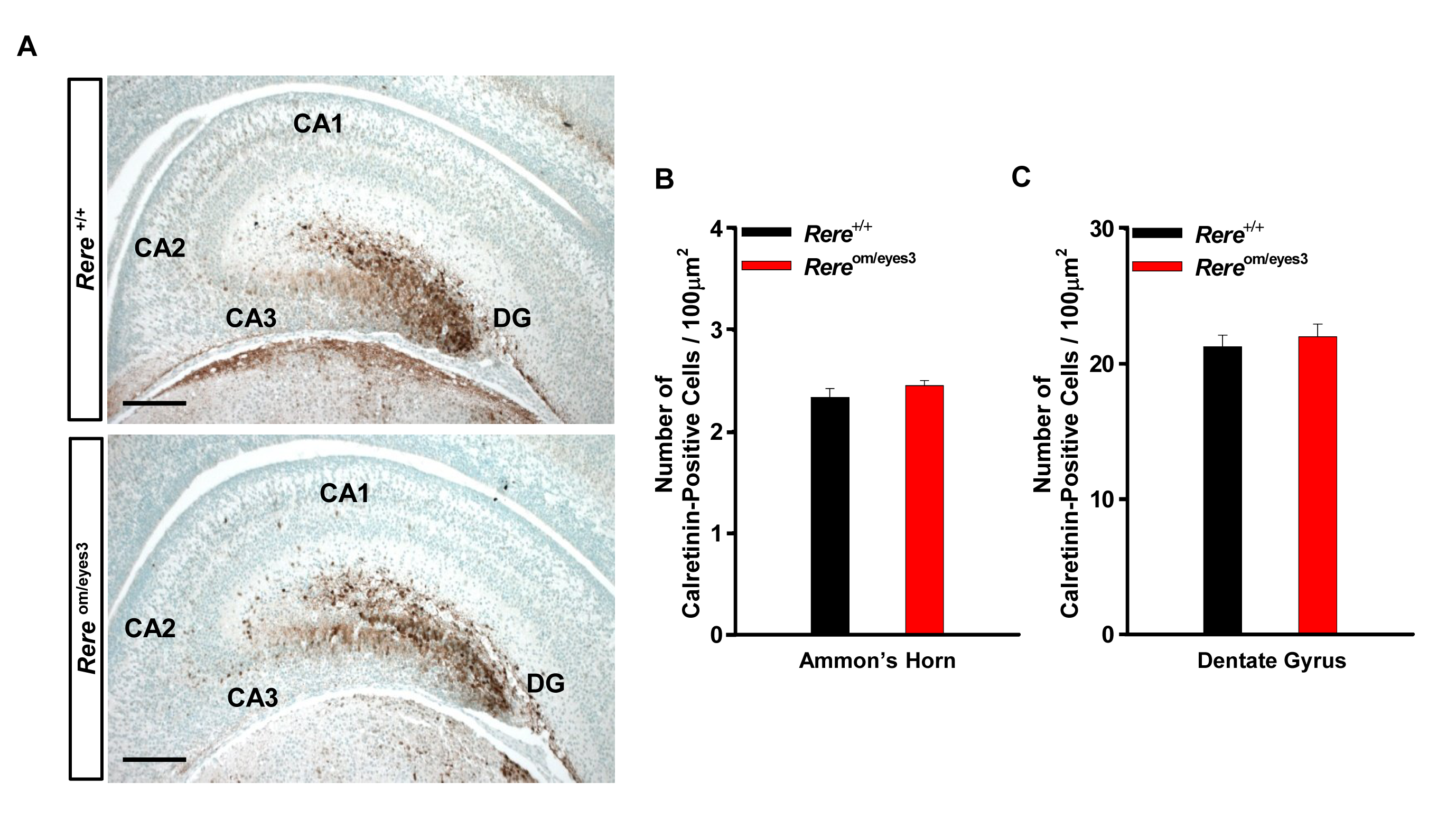

Supplement: Figure S4 — Number of calretinin-positive neurons per area is not changed in the Ammon’s horns or the dentate gyri of Rere om/eyes3 embryos compared to wild-type embryos. A) Coronal sections showing the hippocampus were prepared from wild-type and Rere om/eyes3 embryos and probed with anti-Calretinin antibodies at E18.5. Calretinin-positive cells are abundant in the dentate gyrus. In contrast, a few Calretinin-positive cells are detected in CA3 field. Scale bar indicates 100 µm. B–C) Calretinin-positive cells in the Ammon’s horns, including CA1, CA2, and CA3, and in the dentate gryi of embryos of each genotype were counted and normalized to the area of each region. Number of Calretinin-positive cells per area is not significantly different between the Ammon’s Horns or the dentate gyri of Rere om/eyes3 embryos in comparison with their wild-type littermates (analysis based on twenty slides containing at least three sections for each of three or more embryos). (TIF) [file pone.0057460.s004.tif]

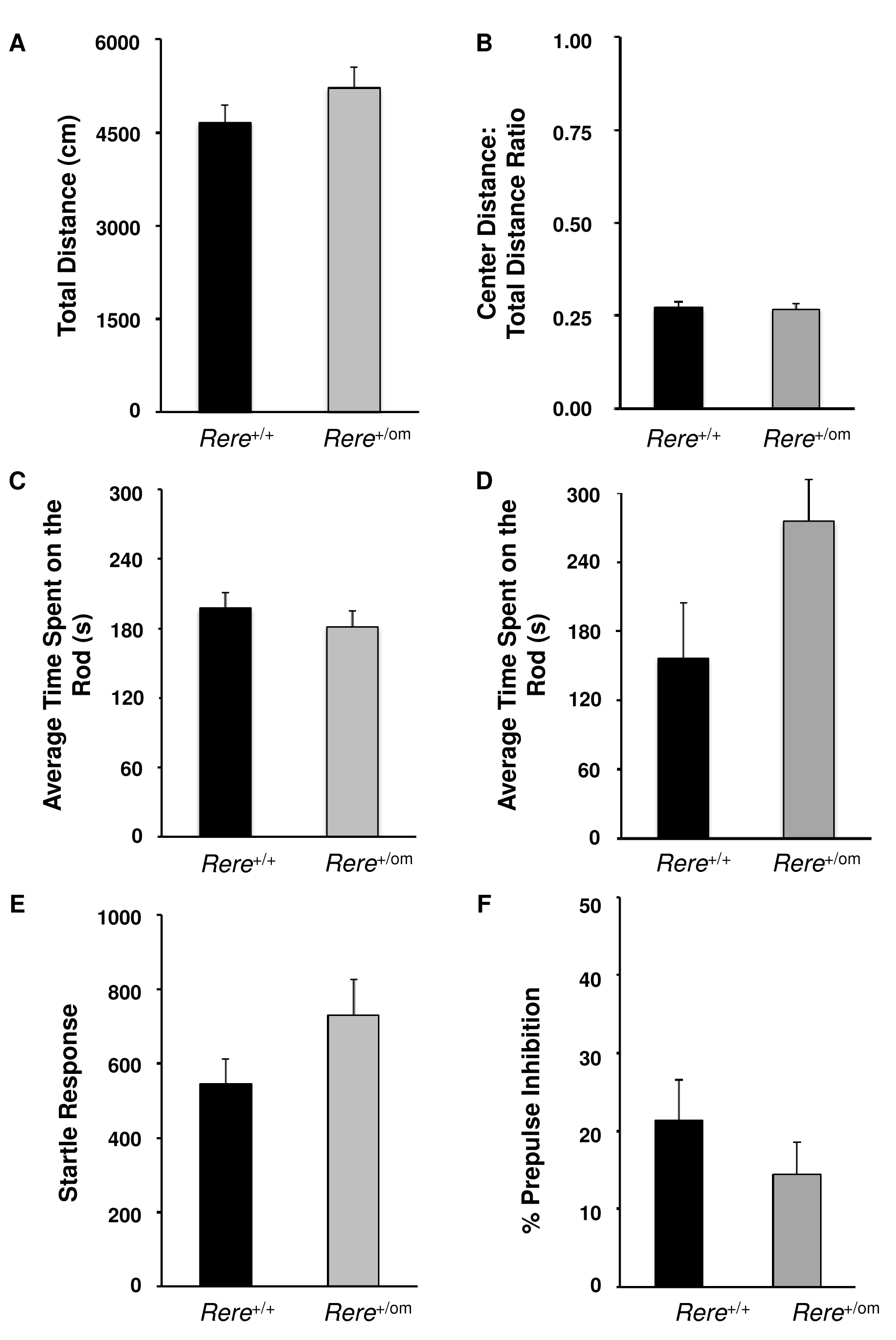

Supplement: Figure S5 — Neurobehavioral analyses do not show a significant difference between wild-type and Rere +/om littermates. A–B) In open field activity testing, no difference was seen in the total distance traveled (panel A; p = 0.247) or the center distance:total distance ratio (panel B; p = 0.832) between wild-type and Rere +/om mice. C–D) In the Rotarod test, no difference was seen between wild-type and Rere +/om mice in the average time spent on the rod (panel C; p = 0.444) or the learning index (panel D; p = 0.054). No differences were seen between Rere +/om mice and their wild-type littermates in tests of acoustic startle (panel E; arbitrary units, p = 0.832) and prepulse inhibition (panel F; p = 0.176). Between 12 and 18 mice per genotype were used for these tests. (TIF) [file pone.0057460.s005.tif]

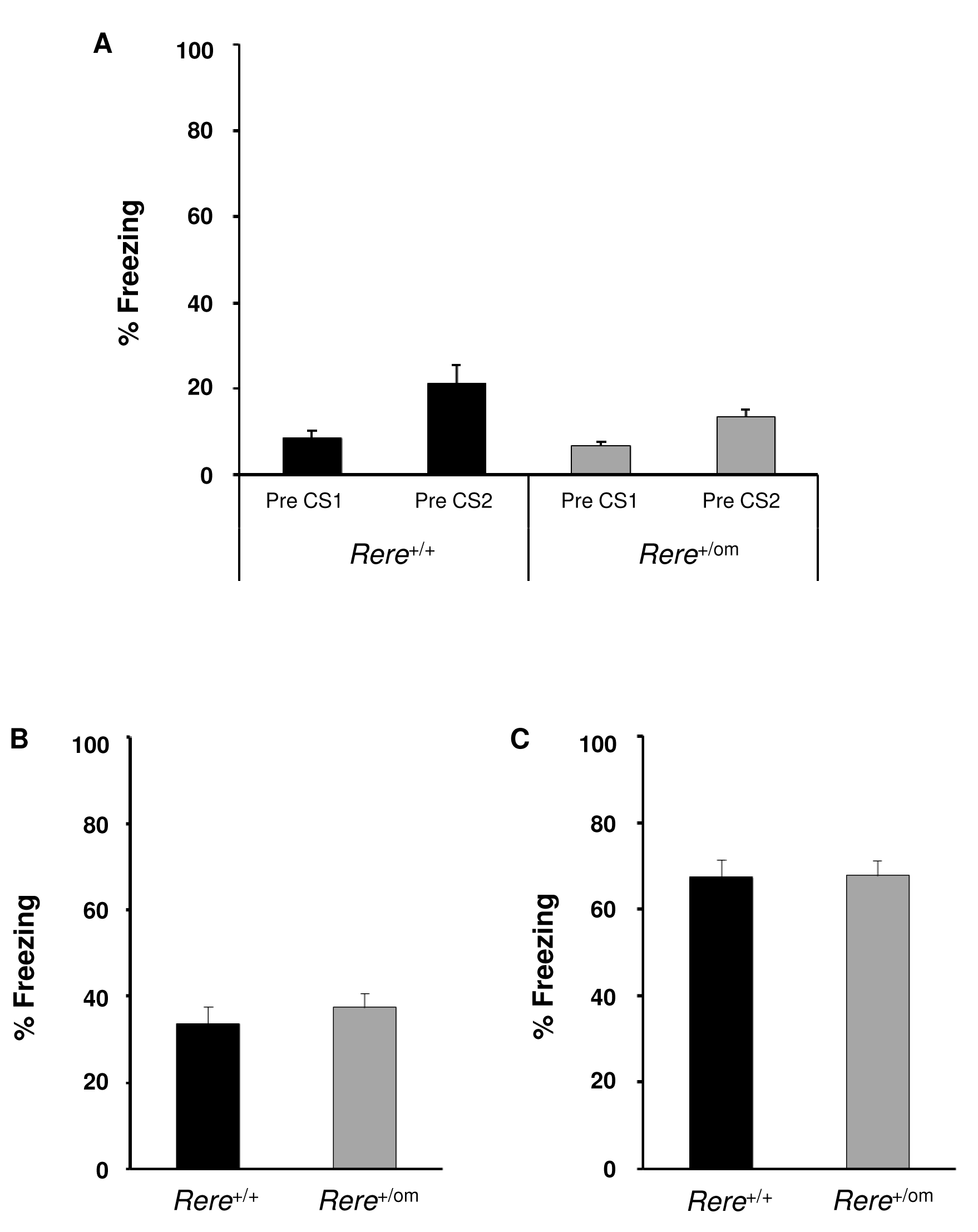

Supplement: Figure S6 — Conditioned fear testing showed no differences between wild-type and Rere om/+ littermates. A) The percent freezing on the training day (day 1) was not found to be significantly different between wild-type and Rere +/om mice (n = 12–18 per genotype). B) The average percent freezing to contextual clues was not different between genotypes (p = 0.469). C) The average percent freezing with the conditioned stimulus minus the percent freezing with the pre-conditioned stimulus (CS - preCS) was not different between genotypes (p = 0.953). (TIF) [file pone.0057460.s006.tif]
